# LILRA5^+^ macrophages drive early oxidative stress surge in sepsis: a single-cell transcriptomic landscape with therapeutic implications

**DOI:** 10.3389/fcimb.2025.1606401

**Published:** 2025-07-28

**Authors:** Peng Xu, Haoze Li, Zuo Tao, Zixuan Zhang, Xiaohuan Wang, Cheng Zhang

**Affiliations:** ^1^ Department of General Surgery, General Hospital of Northern Theater Command, Shenyang, Liaoning, China; ^2^ Department of General Surgery, Beijing Haidian Hospital, Beijing, China

**Keywords:** oxidative stress, sepsis, LILRA5, macrophage, single-cell

## Abstract

**Background:**

In sepsis, oxidative stress (OS) triggers essential adaptive responses and emerging OS-related biomarkers show potential for enhancing sepsis diagnosis and therapy.

**Methodology:**

In this study, we used single-cell datasets and the OS gene set to identify immune cell types with the highest oxidative activity across different sepsis states. Differential expression genes (DEG) between “high state” cells and “low state” cells were screened. High-dimensional weighted gene co-expression network analysis (hdWGCNA), combined with multiple machine learning methods, was used for the selection of hub genes. Expressions of hub genes were then validated. Cell–cell communication and transcription factor analysis were performed later. Real-time quantitative reverse transcription (qRT-PCR) and Western blotting validated expression of LILRA5 in both the cecal ligation and puncture (CLP) model and the lipopolysaccharide-induced sepsis model. Reactive oxygen species (ROS) levels were also detected in THP-1 cells after silencing LILRA5.

**Results:**

In the early stages of sepsis, oxidative activity reaches its peak, with macrophages displaying the highest OS among all cell types. Through the application of the “Quartile method”, all cells were clustered into three states based on OS activity (low, medium, and high). LILRA5, MGST1, PLBD1, and S100A9 were selected as hub genes and significantly upregulated in sepsis. LILRA5 was predominantly expressed in macrophages and was highly expressed in the early stage of macrophage. Specifically, LILRA5^+^ macrophages exhibit the strongest OS. LILRA5 showed a higher expression in both mouse sepsis models and the THP-1 cell after lipopolysaccharide stimulation. Silencing LILRA5 resulted in a significant reduction of ROS in THP-1 cells.

**Conclusion:**

In conclusion, our study has mapped the landscape of OS dynamics in sepsis and found that LILRA5^+^ macrophages in the early stage of sepsis exhibit the highest OS. LILRA5 emerges as a promising gene for modulating macrophage-mediated OS in sepsis.

## Introduction

Sepsis is a life-threatening clinical syndrome characterized by organ dysfunction resulting from a dysregulated host response to infection. As a leading cause of critical illness worldwide, it carries substantial mortality and poses significant therapeutic challenges due to its complex pathophysiology (Liu et al., 2022; Vincent, 2022). Sepsis-induced immunosuppression significantly contributes to adverse outcomes. The progression of sepsis can be categorized into three stages: severe sepsis, septic shock, and multiple organ dysfunction, reflecting the increasing severity and complexity of the condition (Delano and Ward, 2016). Key pathophysiological processes in these stages include oxidative stress (OS), endothelial and mitochondrial dysfunction, and angiogenesis-related factors (Vera et al., 2015; Joffre and Hellman, 2021). OS reflects an imbalance between antioxidant defense mechanisms and free radical production (Neri et al., 2016). In sepsis, OS promotes adaptive responses to bacterial clearance, endothelial repair, and hypoxia. These responses are crucial for the body’s defense mechanisms and tissue homeostasis during infection (Joffre and Hellman, 2021). Therefore, new biomarker categories of OS are drawing attention for the diagnosis and treatment of sepsis.

Single-cell RNA sequencing (scRNA-seq) is highly effective in discerning cell types, states, and lineages (Wang et al., 2022). This powerful technique allows for a deeper understanding of cellular heterogeneity and the intricate dynamics within complex biological systems (Baysoy et al., 2023). Machine learning algorithms, employing both supervised and unsupervised techniques, have demonstrated significant potential for analyzing the underlying relationships within high-dimensional data (Zhang et al., 2023). This approach allows researchers to uncover key genetic factors that may be pivotal in understanding complex biological processes and diseases. In our study, we applied scRNA transcriptome, bulk RNA analysis, and multiple machine learning algorithm to better understand the dynamic changes of OS in sepsis.

In our study, we firstly emphasized the dynamic changes of OS in sepsis. Compared with other cells, macrophages, neutrophils, and dendritic cells (DCs) are three cell types with the most OS in sepsis. Moreover, we also revealed the spatial and temporal heterogeneity on OS in sepsis. OS is more active in the early stage of sepsis. In sepsis, OS proved to be crucial in the initial stage. Next, we also identified multiple cell clusters with different OS activities based on a new classified method, and the high-dimensional weighted gene co-expression network analysis (hdWGCNA) revealed the specific gene modules with high OS activity. Hub genes were then screened by multiple machine learning algorithms and matched with the special cell type. Our results defined a special cluster of macrophage with high OS activity and its driving function in the early state of sepsis. Our findings showed crucial insights into the dynamic changes of OS and the potential immune therapeutics of sepsis.

## Materials and methods

### Data acquisition

The Gene Expression Omnibus (GEO) database (https://www.ncbi.nlm.nih.gov/geo) was used to access the GSE167363 (*n* = 12) and GSE175453 (*n* = 9) scRNA-seq sets. These two datasets (GSE167363 and GSE175453) utilized in this study can be found in **Supplementary Tables 1** and **2**. For bulk RNA-seq data, we utilized the GSE57065 dataset (*n* = 107) as the training dataset and the GSE95233 dataset (*n* = 124) as the test dataset. These two datasets (GSE57065 and GSE95233) utilized in this study can be found in **Supplementary Tables 3** and **4**. We selected OS-related genes (*n* = 807) from the Genecards database (https://www.genecards.org/). Genes are listed in **Supplementary Table 5**.

### scRNA-seq dataset analysis

In the processing of scRNA-seq data, we retained high-quality cells with less than 20% mitochondrial gene content and expressing more than 200 genes. Additionally, we prioritized genes with expression levels ranging from 200 to 7,000 and active in at least three cells. A total of 103,851 eligible cells were retained for further analysis. Subsequently, data integration was performed using the Seurat pipeline. The remaining cells were then scaled and normalized via a linear regression model employing the “Log-normalization” method. Additionally, the top 3,000 highly variable genes were identified using the “FindVariableFeatures” function. Following this, the dimensionality of the scRNA-seq data was reduced using principal component analysis (PCA). To eliminate batch effects among samples, soft k-means clustering was performed with the “Harmony” package. Cell clustering was subsequently carried out using the “FindClusters” function, with the resolution parameter adjusted to 0.6. The annotation of cell clusters was based on genes with high expression levels, genes displaying distinct expression patterns, and established canonical cell markers (Yang et al., 2024).

### Evaluation of OS activity

Five algorithms (including AUCell, Ucell, singscore, ssgsea, and AddModuleScore) were used to evaluate each cell’s OS activity at the single-cell level and determine the overall OS activity. The raw score matrix underwent sequential *Z*-score standardization and Min–Max normalization: initial standardization transformed features to zero-mean and unit-variance distributions, followed by a custom normalization function linearly scaling values to the [0,1] range applied column-wise to ensure feature-wise consistency. The processed matrix was converted into a data frame format, with a composite score derived from row-wise summation of normalized feature values. Based on the quartile method, cells with a score below the 25th percentile were categorized into the low-OS activity state group, those between the 25th and 75th percentiles were classified as those belonging to the “transition state”, and those above the 75th percentile were designated as the high-OS state group. The correlation analysis was used to explore the association between genes and OS activity. The “FindMarkers” function was used to perform differentially expressed gene (DEG) analysis in the upregulated OS group (avg_log2FC > 0.25, *p*_adj_ < 0.05). To assess whether the distribution of cell types differs significantly across the high-, medium-, and low-scoring groups, we constructed contingency tables for each cell type and performed chi-squared tests of independence. For cell types with small sample sizes, robustness was checked with Fisher’s exact test. The resulting *p*-values were adjusted for multiple testing using the false discovery rate (FDR) method.

### High-dimensional weighted correlation network analysis

The hdWGCNA was used to construct a co-expression network using the “hdWGCNA” package. We used the genes expressed in more than 5% of cells, and the “MetacellsByGroups” function was used to construct the metacell gene expression matrix. The soft power was determined by the “TestSoftPowers” function. The “ConstructNetwork” function is employed to build the co-expression network.

### Machine learning algorithms

To identify optimal feature genes associated with overall survival, we performed an integrative analysis of the pre-screened gene set using five distinct machine learning approaches: (1) least absolute shrinkage and selection operator (LASSO) regression, (2) support vector machine–recursive feature elimination (SVM-RFE), (3) Boruta feature selection, (4) random forest (RF), and (5) gradient boosting machine (GBM). This multi-algorithm framework ensured robust identification of hub genes while mitigating model-specific biases. Feature genes were determined by intersecting the outputs of all five algorithms (LASSO, SVM-RFE, Boruta, RF, and GBM), ensuring robust selection. LASSO algorithms eliminated redundant predictors while maintaining discriminative power. SVM-RFE was employed to iteratively prune the feature set by removing the least informative features, thereby improving the model’s predictive performance. The Boruta algorithm assessed the significance of each feature by repeatedly sampling from the original dataset and constructing RFs. Meanwhile, the RF algorithm built multiple decision trees through random sampling and feature selection, and generated predictions via voting or averaging mechanisms. These approaches collectively clarified the correlations and interactions among features. GBM is a powerful ensemble technique that builds models iteratively to minimize a loss function. It is highly effective for a wide range of tasks but requires careful tuning and regularization to avoid overfitting.

### Cell–cell communication and inference of transcription factors

We leveraged CellChat to analyze variations in cell–cell communication modules by integrating gene expression data. Following the standard CellChat pipeline, we employed the default CellChatDB as the ligand–receptor interaction database. Cell type-specific interactions were inferred by identifying overexpressed ligands or receptors within each cell cluster, and validated through the assessment of enhanced ligand–receptor interactions upon their upregulation. This approach ensured robust inference of communication patterns while maintaining alignment with established analytical frameworks. Additionally, the activity of gene regulatory networks was inferred using the R package “Scenic”, while for pseudotime analysis, three algorithms [“Monocyte” (Xu et al., 2024), slingshot (Street et al., 2018), and “CytoTRACE” (Wang et al., 2022)] were used for analysis.

### THP-1 cell cultures

The THP-1 human monocytic leukemia cell line (Type Culture Collection of Chinese Academy of Sciences, China) was cultured in RPMI-1640 medium (Gibco, USA) containing 10% fetal bovine serum (FBS) (Gibco, USA) with 2 mM L-glutamine (Solarbio, China), 1 mM sodium pyruvate (Solarbio, China), 10 mM HEPES (Gibco, USA), and penicillin/streptomycin (Solarbio, China) (50 U/mL each).

The differential protocol of macrophage was according to a previous study (Lai et al., 2023). Cells were maintained at 37°C in a 5% CO_2_ humidified incubator. For lipopolysaccharide (LPS) (Selleck, USA) stimulation experiments, triplicate cultures (1×10^6^ cells/mL/well in 12-well plates) were treated with 10 ng/mL LPS for 24 h. Post-treatment cell pellets obtained by centrifugation (1,000 rpm, 5 min) were divided for parallel processing: RNA extraction using Trizol (TaKaRa Bio Inc., Japan) and protein isolation.

### siRNA transfection

Gene silencing of LILRA5 was achieved using siRNA technology with Lipofectamine™ 2000 (Invitrogen, USA). Specifically, the siRNA duplexes targeting the sequence were commercially synthesized (Sangon Biotech, Shanghai). When cells reached 30%–50% confluency, transfection was performed with either LILRA5-targeting siRNA or non-targeting control (NC) using the Lipofectamine™ 2000 system. Following 6 h of transfection, cells were processed for downstream analyses.

### Real-time quantitative reverse transcription detecting system

RNA concentration was measured using the NanoDrop 2000 spectrophotometer (Thermo Fisher Scientific, MA, USA). For reverse transcription, the SuperScript First-Strand Synthesis System RT-PCR kit (TaKaRa Bio Inc., Japan) was employed to synthesize complementary DNA (cDNA) from RNA. Quantitative PCR was conducted using the GoTAq qPCR Master Mix (Promega, USA) on the Rotor-Gene Q PCR detection system (Qiagen, Germany). A 10-µL reaction mixture was subjected to thermal cycling, beginning with an initial denaturation at 95°C for 10 min. This was followed by 40 cycles of amplification, consisting of 95°C for 5 s, 60°C for 30 s, and 72°C for 30 s. Subsequently, gene expression levels were quantified using the2^–ΔΔCt^ method. Primer sequences are listed in **Table 1**.

**Table 1:** Sequences of primers.

Primer	Sequence
LILRA5 (homo) -Forward	5’-TCACGGCTGAGATTCGACAG-3’
LILRA5 (homo) -Reverse	5’-CCTGCGAGAGCCATAGCATC-3’
LILRA5 (mouse) -Forward	5’-CGGAAGGGAATCCGCACAA-3’
LILRA5 (mouse) -Reverse	5’-CACCTCACATGAGATGGTCAC-3’
GAPDH (homo) -Forward	5’-GGAGCGAGATCCCTCCAAAAT-3’
GAPDH (homo) -Reverse	5’-GGCTGTTGTCATACTTCTCATGG-3’
GAPDH (mouse) -Forward	5’-AGGTCGGTGTGAACGGATTTG-3’
GAPDH (mouse) -Reverse	5’-GGGGTCGTTGATGGCAACA-3’

### Western blotting

THP-1 cells were seeded at a density of 1×10^6^ cells/mL in triplicate wells of 12-well plates. Experimental groups were stimulated with LPS (10 ng/mL) for 30 min, while control groups were treated with phosphate-buffered saline (PBS) (Thermo Fisher, USA). Proteins were extracted from THP-1 cells using RIPA (Solarbio, China) and phenylmethanesulfonyl fluoride (PMSF) (Boster, USA). After polyacrylamide gel electrophoresis, proteins were transferred to a polyvinylidene fluoride (PVDF) membrane (Vazyme, China). The membrane was blocked, incubated overnight with a primary antibody against LILRA5 and β-actin (Proteintech, USA), and then incubated with a secondary antibody for 1 h. Results were detected using a Western Blot Imaging System (4000R, CareTream, USA).

### Establishment of the sepsis mouse model

Cecal ligation and puncture (CLP) models: Eight-week C57 mice were put into slumber after inhaling isoflurane, and a 22-gauge needle was used once to puncture a stump in order to release stool. The abdomen was then closed after the cecum was moved intraabdominally. Saline (0.2 mL) was injected via the abdomen for fluid resuscitation. Sham-operated mice were not ligated or punctured. Four hours following the procedure, the mice were put back in their cages and slaughtered. We induced sepsis in mice through intraperitoneal injection of LPS at a dose of 20 mg/kg, with some mice receiving PBS as a control group. This method established an LPS-induced sepsis model.

### Reactive oxygen species detection assay

Intracellular ROS levels were quantified in THP-1 cells following LPS stimulation (10 ng/mL) or vehicle treatment (0.1% BSA). After a 24-h incubation at 37°C in a humidified 5% CO_2_ incubator, ROS induction was assessed using the ROS assay kit (Beyotime, China), which employs the cell-permeant fluorogenic probe 2′-7′-dichlorofluorescein diacetate (DCFH-DA). This probe is hydrolyzed by cellular esterases into DCFH carboxylate, which is subsequently oxidized by intracellular ROS into the fluorescent 2′-7′-dichlorofluorescein (DCF). For the assay, cells were loaded with 15 μM DCFH-DA in culture media, incubated at 37°C for 30 min, and analyzed by flow cytometry without washing. The intracellular ROS levels were expressed as mean fluorescence intensity (MFI) values.

### Statistical analysis

Data handling and visualization were performed using R 4.2.0. Statistical significance was determined using a two-tailed test, with *p*-values less than 0.05 considered significant.

## Results

### The scRNA profiling of sepsis

**Figure 1:**
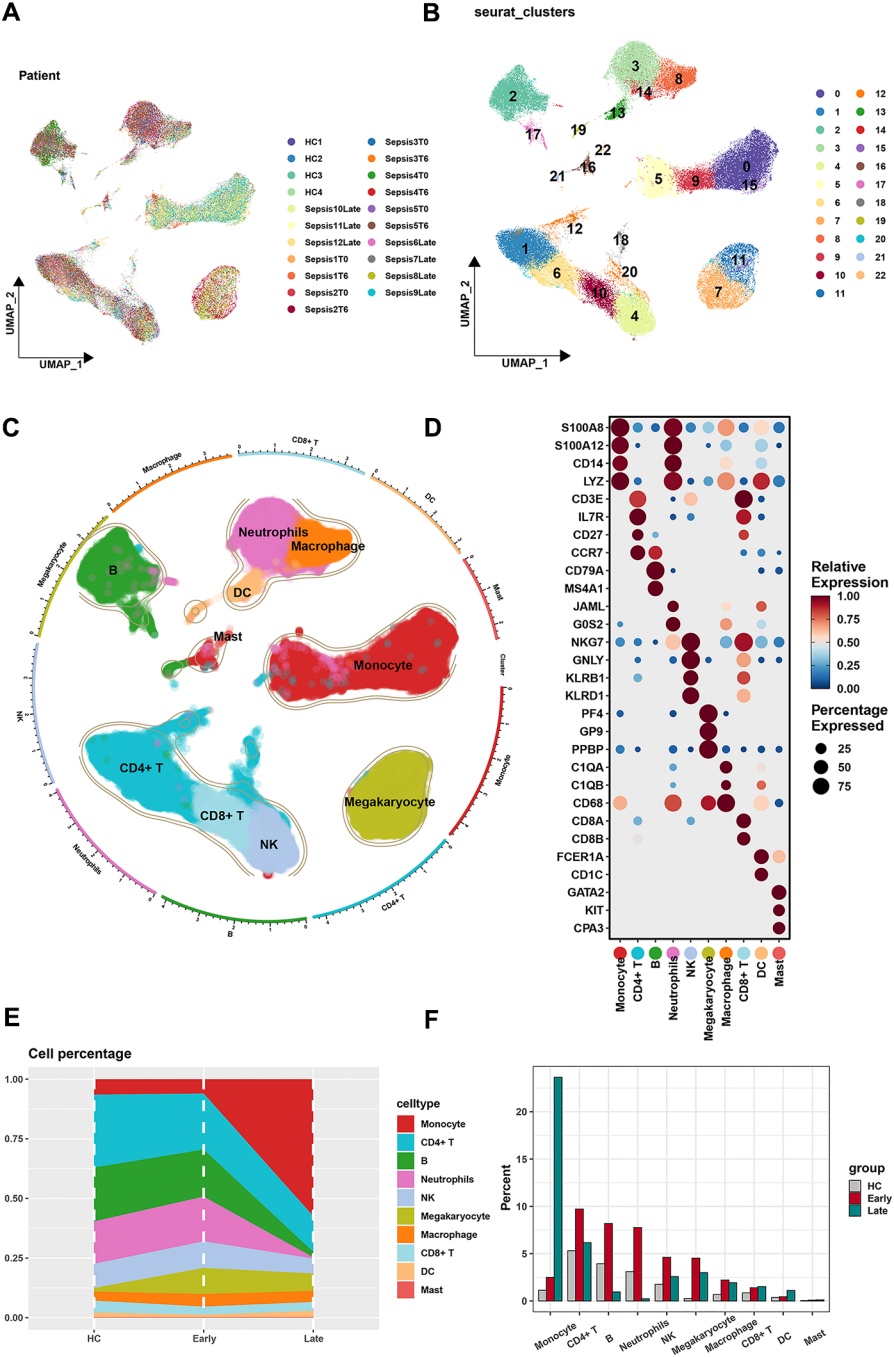
Screening of single-cell data. **(A)** The results of PCA revealed a relatively stable cell distribution across all analyzed samples with low sensitivity to batch effects. **(B)** The results of UMAP indicated that all cells were meticulously classified into 23 clusters. **(C)** Based on classic marker genes, the data were manually annotated into 10 different cell types. **(D)** Expression of classic marker genes in 10 different cell types. **(E, F)** Cell percentages of 10 cell types originated from samples of different stages in sepsis.

Our study comprised a total of 21 samples, each demonstrating a uniform cell distribution. Given the consistency observed across all samples, we infer that batch effects likely had minimal influence on the results (**Supplementary Figure 1**). Distributions of each sample on the single-cell level were visualized by the UMAP algorithm (**Figure 1A**). Based on single-cell analysis, cells were categorized into 23 different clusters (**Figure 1B**). Diverse cell types (monocytes, CD4^+^ T cells, B cells, neutrophils, NK cells, megakaryocytes, macrophages, CD8^+^ T cells, DCs, and mast cells) were unveiled according to different markers (**Figure 1C**). The densities of markers were shown (monocyte: S100A8, S100A12, CD14, and LYE; CD4^+^ T cell: CD3E, IL7R, CD27, and CCR7; B cell: CD79A and MS4A1; neutrophil: JAML and GOS2; NK cell: NKG7, GNLY, KLRB1, and KLRD1; megakaryocyte: PF4, GP9, and PPBP; macrophage: C1QA, C1QB, and CD68; CD8^+^ T cell: CD8A and CD8B; DC: FCER1A and CD1C; mast cell: GATA2, KIT, and CPA3) (**Figure 1D**). The density of each marker is shown in **Supplementary Figure 2**. The features of markers are shown in **Supplementary Figure 3**. Corresponding proportions of 10 cell types in different states of sepsis (health control, the early stage, and the late stage) were presented (**Figures 1E, F**). Gene ontology (GO) analysis revealed potential functional mechanisms and offered insights into the biological roles of 10 heterogeneous cell populations (**Supplementary Figure 4**).

### The heterogeneity of OS activity in each cell cluster in sepsis

**Figure 2:**
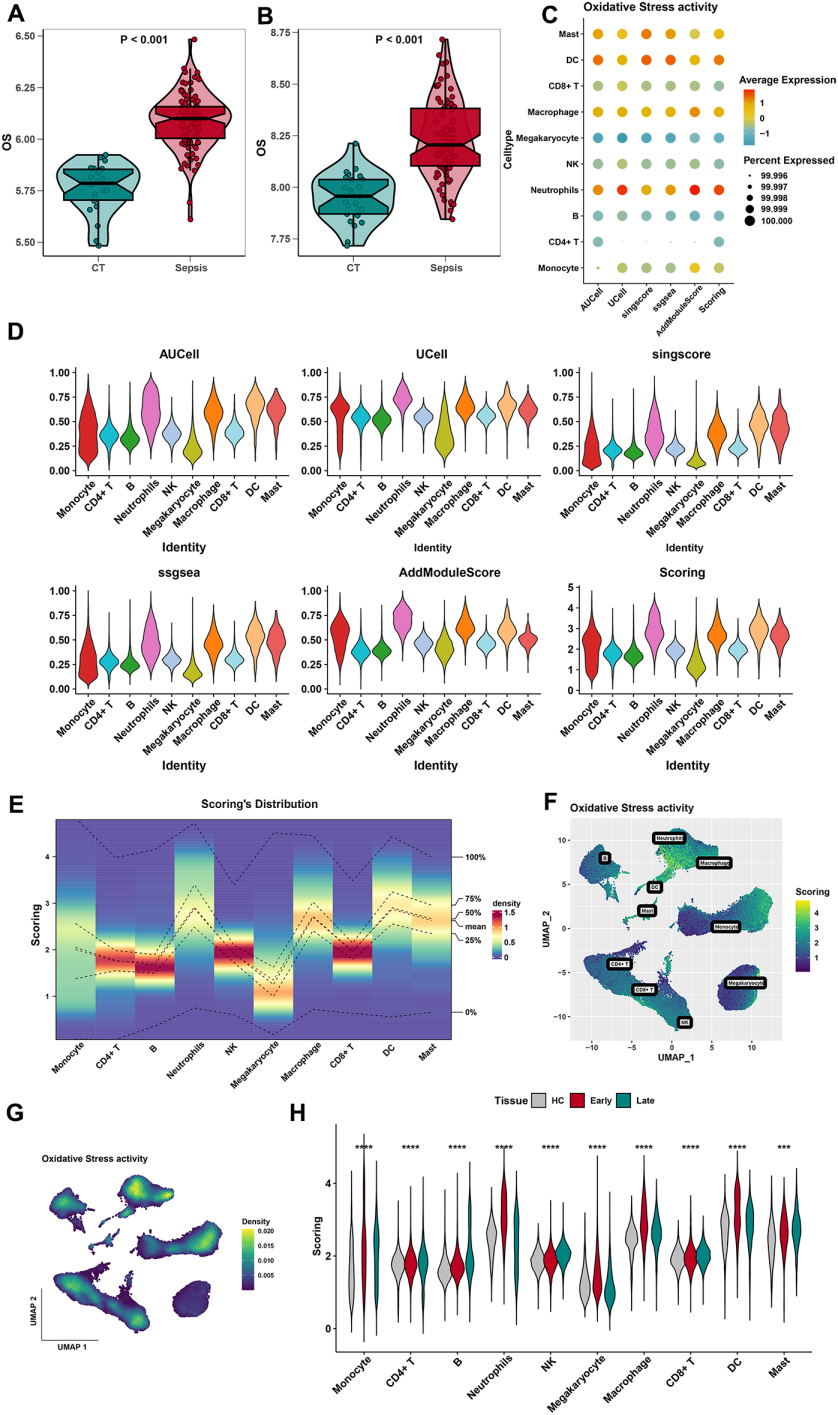
Identification of the most relevant cell types for OS activity. **(A)** Differences in the oxidative stress in different groups (control and sepsis) of the training dataset. **(B)** Differences in the oxidative stress in different groups (control and sepsis) of the test dataset. **(C, D)** The results of AUCell, Ucell, singscore, ssgsea, and AddModuleScore algorithms showed that mast cells, DCs, macrophages, and neutrophils had the highest aggregation activity, while other cells had relatively lower activity. **(E)** Distribution of OS score in 10 cell types. **(F)** Scores of oxidative stress activities in 10 types of cells by the UMAP plot. **(G)** The density of oxidative stress activities in 10 types of cells by the UMAP plot. **(H)** Distribution of OS score in 10 cell types of three stages in sepsis. ***, p<0.001; ****, p<0.0001.

A total of 807 OS-related genes were downloaded from GeneCards with a relevance score ≥ 7. The OS was elevated in sepsis in both training and test sets (**Figures 2A, B**). Notably, the expression levels of the OS genes were significantly higher in both sepsis samples than in the control tissues. **Supplementary Figure 5A** shows the expression of 807 OS-related genes in three different stages of sepsis (healthy control, early stage, and late stage). The expression of the OS-related genes was significantly higher in the early stage of sepsis. Five algorithms (including AUCell, UCell, singscore, ssgsea, and AddModuleScore) were applied to evaluate the index of OS (**Figures 2C, D**). The expression of 807 OS-related genes in 10 types of cells is shown (**Figure 2E**; **Supplementary Figure 5B**). According to the OS score, neutrophils, macrophages, DCs, and mast cells are four cell types with the most OS. Scores and density of OS-related genes in 10 types of cells are also visualized in **Figures 2F, G**. Moreover, OS scores of 10 cell types were also visualized in different stages of sepsis (**Figure 2H**). In contrast, neutrophils, macrophage, and DCs are three cell types with the most OS activity in the early stage of sepsis. In conclusion, neutrophils, macrophages, and DCs may play a potential role in response to sepsis.

### Identification of cells and gene modules with different OS states

**Figure 3:**
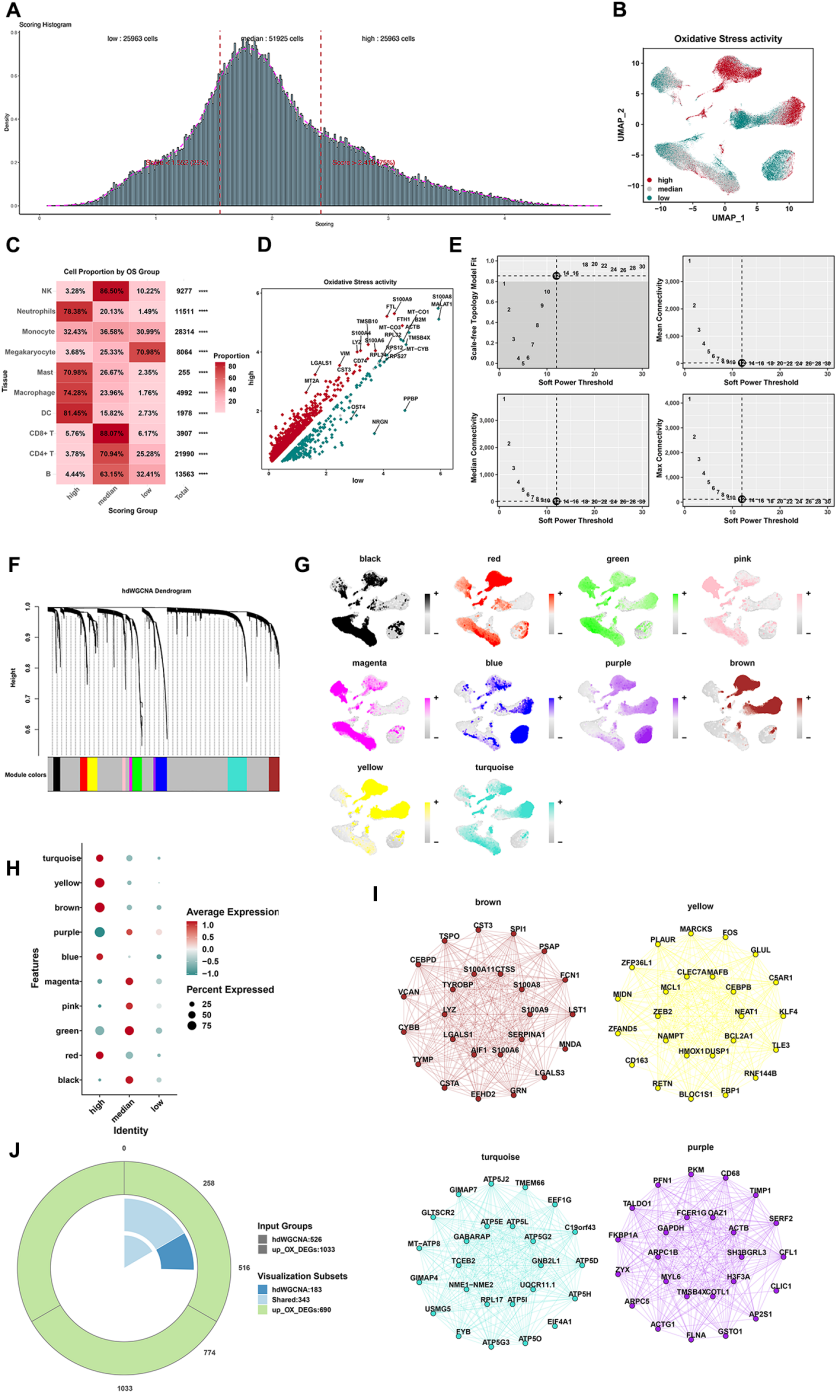
Identification of cells and gene modules with different OS activity states. **(A)** According to the median of the total OS score, cells were classified as high-OS cells (top 25%) and low-OS cells (low 25%) according to the median of the total OS activity (25%–75%). **(B)** Cell distributions in low, medium, and high state by the UMAP plot. **(C)** The cell proportions in the low, medium, and high state. **(D)** The results of DEG analysis of OS by the volcano map (****p < 0.0001). **(E)** The top left panel depicted the soft power threshold for choosing a scale-free topology model fit greater than or equal to 0.9. The other three panels showed the mean, median, and max connectivity of the topological network respectively when different minimum soft thresholds are chosen, reflecting the connectivity of the network. The average connectivity of the topological network is most stable at the lowest soft threshold equal. **(F)** Ten modules were identified as shown in the hdWGCNA dendrogram. **(G)** Expression density of each module in the UMAP plot. Yellow indicates the highest activity score of the module in corresponding cells. **(H)** The bubble plot displayed the scores obtained by 10 modules in three groups with high, medium, and low OS scores. **(I)** The results of PPI analysis of the four gene modules (brown, yellow, turquoise, and purple). **(J)** Venn diagram for screening 343 shared genes between DEGs and hdWGCNA.

To explore the cell type with the most OS, this study defined three cell groups by the quartile method according to the OS scores (**Figure 3A**). The top 25% cells were defined as the high-OS cells (25,963 cells with OS score > 2.419). The bottom 25% cells were defined as the low-OS cells (25,963 cells with OS score < 1.552). The medium cells were defined as those belonging to the “transition state”. Macrophages, neutrophils, DCs, and mast cells were four types of cells with the most OS in response to infection (**Figures 3B,C**). Moreover, we also define a new cluster of upregulation genes between the high-OS cells and the low-OS cells (**Figure 3D**). This new cluster of genes may have a real effect in those cells in response to sepsis.

We next applied hdWGCNA to further investigate the characteristics and functions of genes. After weighing median connectivity, mean connectivity, and scale-free topology model fit, a power value of 12 was selected (**Figure 3E**). Ten modules were generated accordingly (**Figure 3F**). Ten gene modules were obtained and the top huh gene was presented following the hdWGCNA pipeline (**Supplementary Figure 6A**). Correlations between every two modules are shown in **Supplementary Figure 6B**. Among 10 modules, the brown module showed the most positive correlation with macrophages and monocytes (**Figure 3G**). Genes in brown, yellow, turquoise, and purple modules showed a higher expression in high-OS cells (**Figure 3H**). Protein–protein interaction (PPI) networks of brown, yellow, turquoise, and purple modules were visualized (**Figure 3I**), while PPI networks of six other modules were also visualized (**Supplementary Figure 6C**). A total of 343 shared genes between OS-related genes and screened genes from hdWGCNA may be the real OS-related genes in sepsis (**Figure 3J**). Disease oncology (DO) and GO analysis of these hub genes were visualized (**Supplementary Figures 7A, B**).

### Machine learning algorithms reveal the OS-related model and hub features

**Figure 4:**
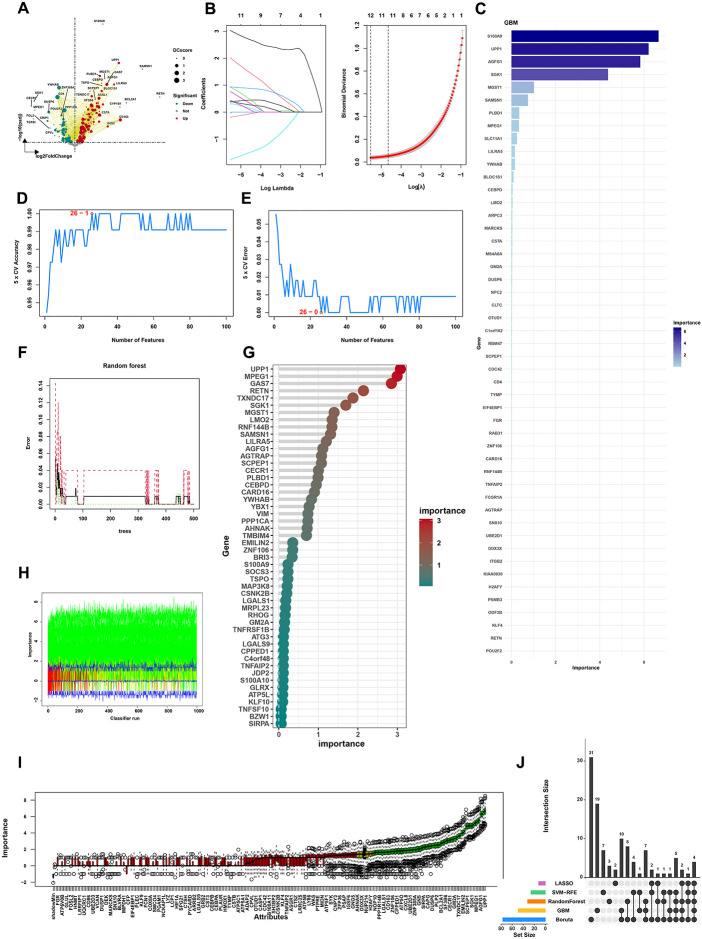
Machine learning algorithms reveal the OS-related model and hub features. **(A)** DEG analysis identified 292 DEGs in 343 shared genes. **(B)** The results of the LASSO algorithm. **(C)** The results of the GBM algorithm. **(D)** The accuracy of the SVM-RFE algorithm. **(E)** The error of the SVM-RFE algorithm. **(F, G)** The results of the Random Forest algorithm. **(H)** The changes in the importance scores of the variables in the Boruta algorithm. **(I)** Number of iterations in the Boruta algorithm. **(J)** The results of the Venn diagram of machine learning algorithms.

To identify hub genes in the 343 shared genes, the differential expression analysis showed 292 DEGs (**Figure 4A**). The selected genes were then integrated into various machine learning algorithms, such as LASSO regression, SVM-REF, Boruta, GBM, and RF. We performed LASSO regression on these genes, which reduced the gene number to 12 (**Figure 4B**). The GBM algorithm screened 49 feature genes (**Figure 4C**). SVM-REF screened 26 feature genes. The accuracy and error of the SVM-FRE showed a good performance (**Figures 4D, E**). The RF algorithm screened 32 feature genes (**Figures 4F, G**). Moreover, the Boruta algorithms also found 77 hub genes (**Figures 4H, I**). Above all, the Venn diagram screened four shared genes (LILRA5, MGST1, PLBD1, and S100A9) as hub genes (**Figures 4J**).

### Validation of hub features in 10 cell types

**Figure 5:**
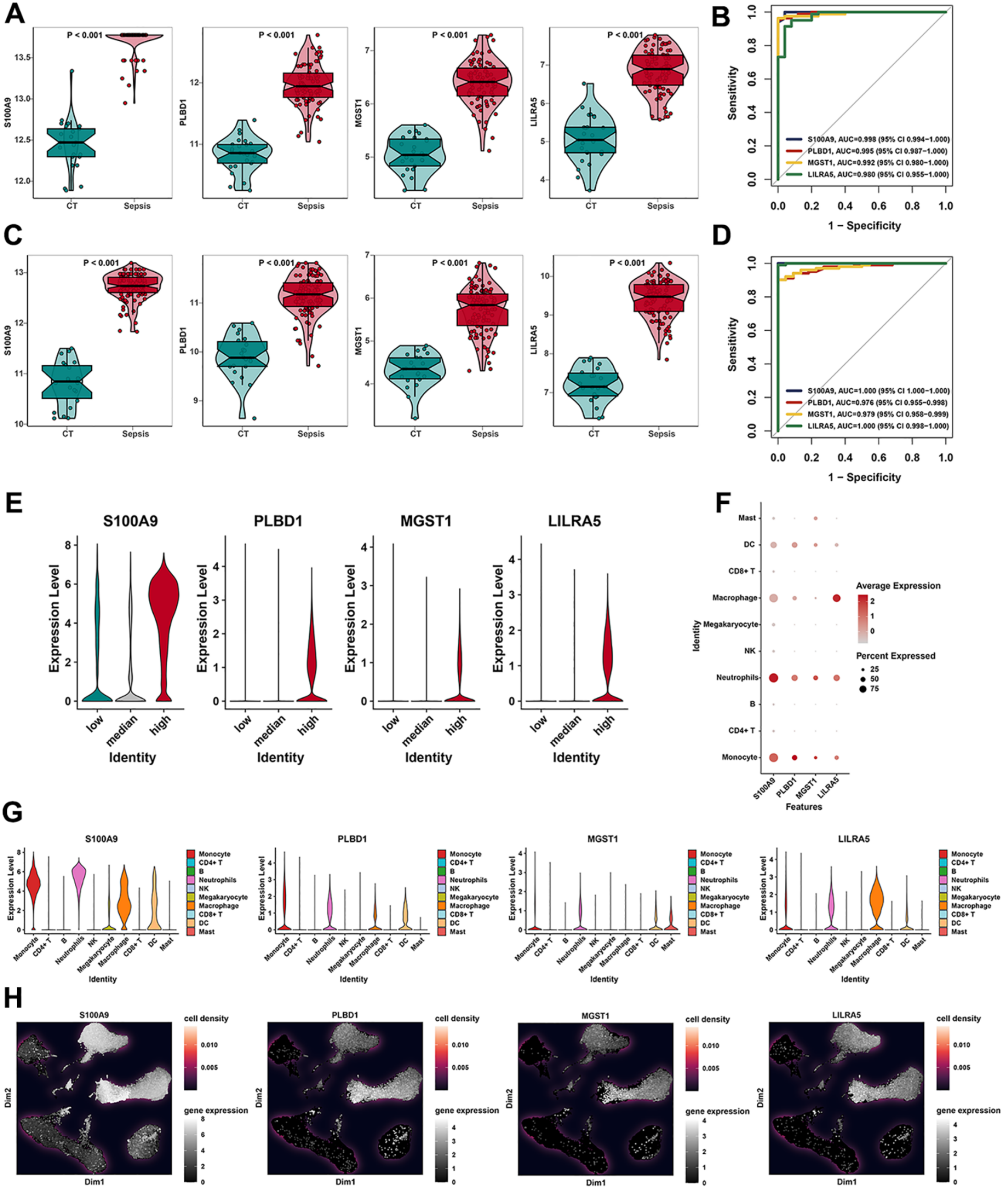
Validation of hub features in 10 cell types. **(A)** Box plots revealed the expression of four feature genes (S100A9, PLBD1, MGST1, and LILRA5) in the training dataset. **(B)** ROC curves of our feature genes (S100A9, PLBD1, MGST1, and LILRA5) in the training dataset. **(C)** Box plots revealed the expression of four feature genes (S100A9, PLBD1, MGST1, and LILRA5) in the testing dataset. **(D)** ROC curves of four feature genes (S100A9, PLBD1, MGST1, and LILRA5) in the testing dataset. **(E)** The violin plot showed the expression of S100A9, PLBD1, MGST1, and LILRA5 in low-, medium-, and high-OS groups. **(F)** Expression of S100A9, PLBD1, MGST1, and LILRA5 in 10 cell types by the bubble plot. **(G, H)** The results of UMAP indicated the expression of four feature genes in 10 cell types.

To evaluate the reliability and accuracy, we evaluated the four feature genes (LILRA5, MGST1, PLBD1, and S100A9) in the bulk level. In the training dataset, the expression of LILRA5, MGST1, PLBD1, and S100A9 was upregulated significantly in the sepsis group compared to the normal group (**Figure 5A**). Moreover, receiver operating characteristic (ROC) curves were used to evaluate the diagnostic potential of the four genes. Area under the ROC curves for LILRA5, MGST1, PLBD1, and S100A9 were 0.980, 0.992, 0.995, and 0.998, respectively (**Figure 5B**). Moreover, in the test datasets, the expression of LILRA5, MGST1, PLBD1, and S100A9 was significantly elevated in the sepsis group (**Figure 5C**). In the test dataset, the areas under the ROC curve (AUCs) for LILRA5, MGST1, PLBD1, and S100A9 were 1.000, 0.979, 0.976, and 1.000, respectively (**Figure 5D**). To evaluate the correlation between four genes and OS, we utilized the bulk data for further analysis. Correlations between each of the two genes and OS-related genes in the training dataset and the test dataset were revealed (**Supplementary Figures 8A, B**). In the training and test dataset, OS activity was elevated in sepsis samples (**Supplementary Figures 8C, D**). These four genes were also significantly upregulated in the group with high OS activities (**Figure 5E**). Correlation of each of the four genes and the OS gene set in the training dataset and the test dataset revealed that these four genes were all positively associated with OS (**Supplementary Figures 8E, F**). Expression features of four genes in different cell types were also delineated at the single-cell level. The results demonstrated that S100A9, PLBD1, MGST1, and LILRA5 were highly expressed in DCs, macrophages, neutrophils, and monocytes (**Figure 5F**). Moreover, expressions of four genes in 10 types of cells were visualized (**Figure 5G**) and the results of UMAP also revealed the expression density of four genes (**Figure 5H**). Moreover, four genes were all positively associated with the OS activity at the single-cell level (**Supplementary Figure 7G**). In our study, we chose LILRA5 for further study. As LILRA5 was significantly upregulated in the macrophage, LILRA5 may play an important role in the OS activities in macrophages.

### Cell–cell communication and transcription factor analysis

**Figure 6:**
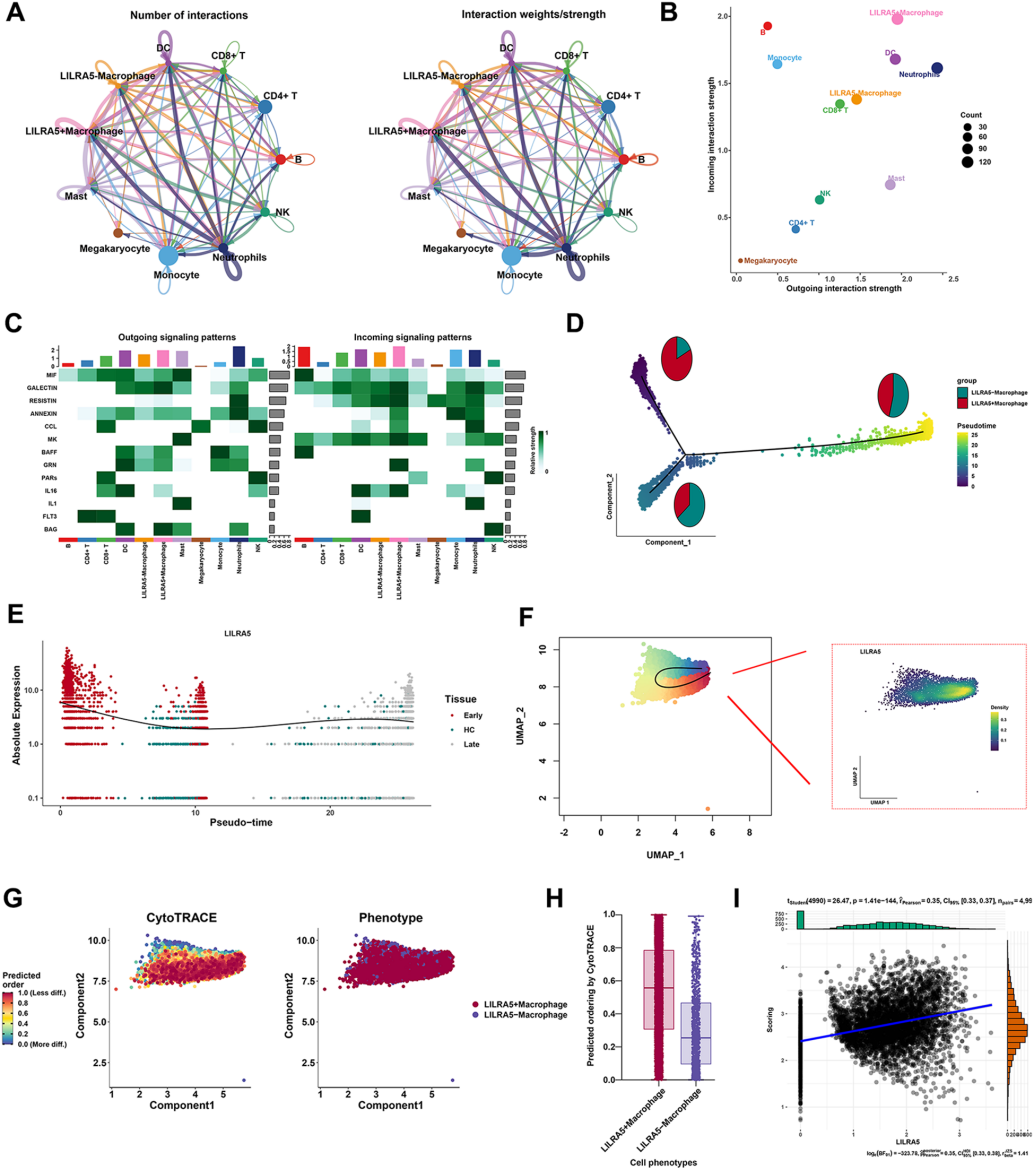
Cell–cell communication and transcription factor analysis. **(A)** Cell chat analysis of all cell types. Both interaction numbers and interaction strengths are shown. **(B)** Scatter plot indicates the differences of incoming and outgoing interaction strengths among all cell types. **(C)** Top cell cytokines were shown in the heatmap across all cell types in sepsis. **(D)** As the quasi-temporal process unfolds, the proportions of LILRA5^+^ macrophage and LILRA5^−^ macrophage differentiated synchronously. **(E)** The relative expression of LILRA5 in the pseudotemporal analysis. **(F)** The slingshot analysis combined with the expression of LILRA5 by the UMAP plot. **(G)** The CytoTRACE analysis of LILRA5^+^ macrophage and LILRA5^−^ macrophage. **(H)** The predicting ordering of CytoTRACE by the boxplot. **(I)** The correlation analysis between LILRA5 expression and cytoTRACE scores.

To explore the function of LILRA5 in macrophages, cell–cell communication and transcription factor analysis were performed. The overall communication ability of LILRA5^+^ macrophage and LILRA5^−^ macrophage with other cell types is shown in **Figure 6A**. Interactions between each cell type and other cell types are visualized in **Supplementary Figure 9**. Interestingly, the outgoing interaction strength showed that the secretory ability of LILRA5^+^ macrophage was stronger compared with other cells (**Figure 6B**), including MIF, GALECTIN, and RESISTIN signaling (**Figure 6C**). Ligand–receptor analysis indicated that MIF-(CD74^+^CXCR4) and MIF-(CD74^+^CD44) were more activated in the signaling from LILRA5^+^ macrophage to B cells and DCs (**Supplementary Figure 10A**). Moreover, RETN-CAP1 signaling was most significant between neutrophils to LILRA5^−^ macrophage and neutrophils to LILRA5^+^ macrophage (**Supplementary Figure 10B**). Compared with LILRA5^−^ macrophage, LILRA5^+^ macrophage has a much higher proportion in the early stage of macrophage (**Figure 6D**). The pseudotime and the slingshot analysis showed that LILRA5^+^ was highly expressed in the early stage of macrophage (**Figures 6E, F**). Moreover, the CytoTRACE analysis was also performed. LILRA5^+^ macrophage had higher CytoTRACE scores, which indicated that this cell type appears in the earlier stage (**Figures 6G, H**). LILRA5^+^ macrophages have a higher tendency towards an “undifferentiated” state based on the predicted scores. Moreover, expression of LILRA5 correlated positively with cytoTRACE scores (**Figure 6I**). The results supported the conclusion that LILRA5 expression is higher in the early stages of development.

### Validation of LILRA5 in macrophages in sepsis

**Figure 7:**
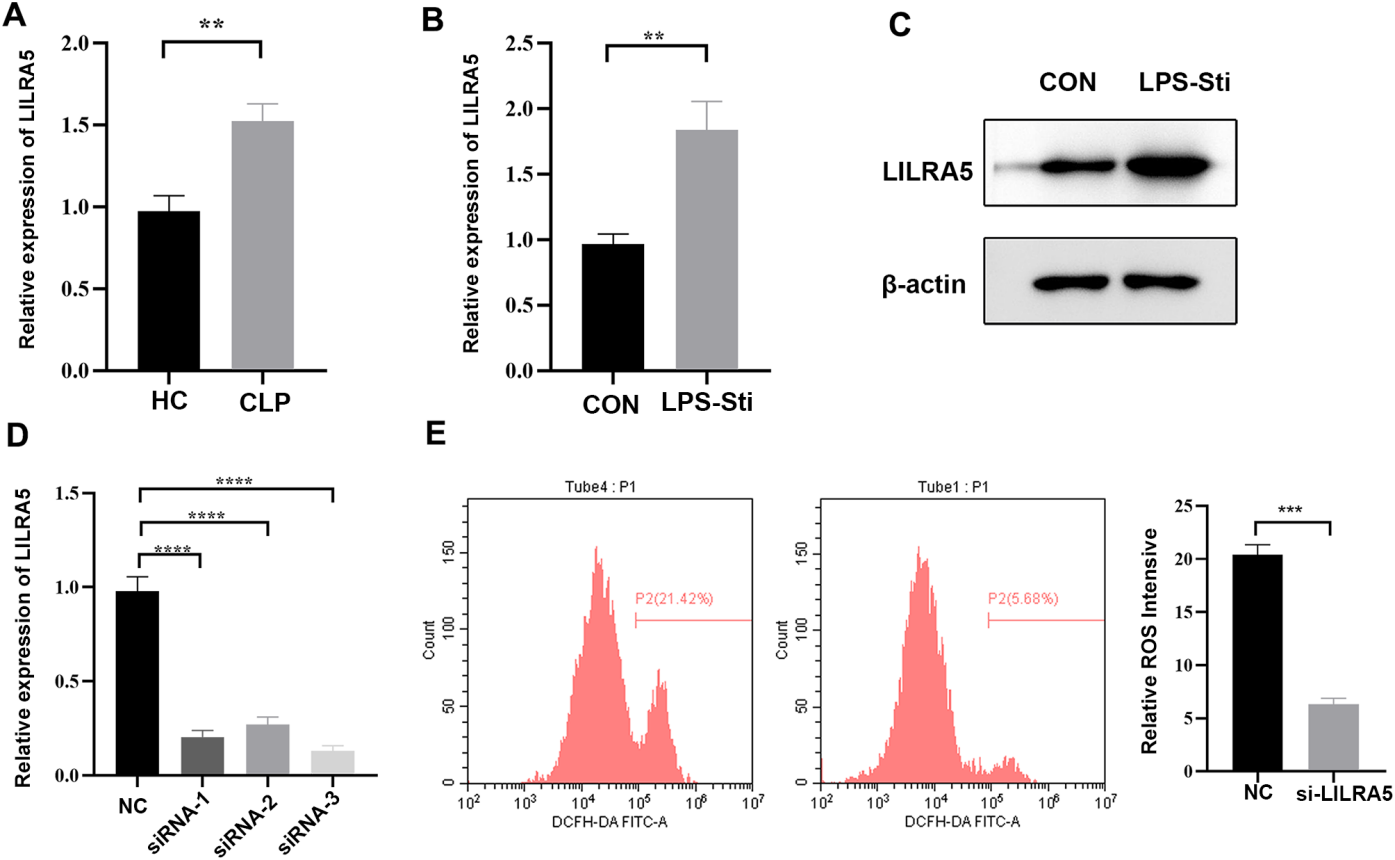
Validation of LILRA5 in macrophages in sepsis. **(A)** mRNA expression of LILRA5 in the blood of the CLP mouse model by qRT-PCR. **(B)** mRNA expression of LILRA5 in the blood of the LPS-stimulated sepsis mouse model by qRT-PCR. **(C)** mRNA expression of LILRA5 after siRNA transfection by qRT-PCR. **(D)** The protein level of LILRA5 in THP-1 cells by Western blotting. **(E)** DCFH-DA probes were used to detect total ROS levels in THP-1 cells. After the DCFH-DA probe was pre-loaded into the THP-1 cells, the THP-1 cells were stimulated with LPS, and the fluorescence signal intensity of the cells was detected (**p < 0.01,***p < 0.001,****p < 0.001).

To validate the expression of LILRA5 in sepsis, we established the CLP mouse model, and the blood of mice was derived. Real-time quantitative reverse transcription (qRT-PCR) showed that LILIR5 was significantly upregulated in sepsis (**Figure 7A**). Our study proved that LILRA5 was mostly expressed in macrophages and potentially regulated OS activity. qRT-PCR showed that the mRNA expression of LILRA5 was higher following the stimulation of LPS in THP-1 cells (**Figure 7B**). The protein level of LILRA5 was higher (**Figure 7C**). To explore the role of LILRA5 in regulating OS in macrophage, we first silenced LILRA5 expression by siRNA (**Figure 7D**). After silencing the LILRA5 in THP-1 cells, the ROS levels were then measured. The result showed that the ROS level was lower when the LILRA5 was silenced (**Figure 7E**). In conclusion, LILIRA5 regulated OS of macrophages positively in sepsis.

## Discussion

Sepsis is a life-threatening condition characterized by dysfunction of multiple organs and dysregulated innate and inflammatory responses of the host to infection (Yang et al., 2022). The fundamental macro- and microcirculatory perturbations induce hypoxia, manifested by lactate accumulation and the eventual failure of organs (Kuebart et al., 2023). Production of IL-1β and TNF-α under stress targeted immune cells (macrophages, neutrophils, and endothelial cells), inducing secretion of ROS and enhancing the inflammatory cascade response (Ouyang et al., 2024). ROS, as critical intracellular signaling molecules, are integral to the development and progression of inflammatory conditions. A previous study showed that ROS production was tightly associated with intracellular bacteria clearance (West et al., 2011). Stimulation of LPS was found to induce ROS production, which leads to the formation and activation of Nod-like receptor family pyrin domain-containing 3 (NLRP3) inflammasome (Sanlioglu et al., 2001; Xu et al., 2025). Activation of NLRP3 inflammasomes is associated with various organ (central nervous system, cardiovascular system, respiratory system, gastrointestinal system, and renal system) damages in sepsis (Bai et al., 2020; Shi et al., 2021). Moreover, NLRP3 inflammasomes also co-localize with mitochondria and are responsible for producing mitochondrial reactive oxygen species (mtROS) (Park et al., 2013). Another study showed that inhibition of ROS production increases the survival rate of CLP mice (Gu et al., 2018). The accumulation of excess ROS leads to OS (Chen et al., 2024). As biological processes of OS play a vital role in sepsis, relative studies are rare. The interaction between OS and immune cells needs to be further studied.

The central mechanism in sepsis involves immune dysfunction, with macrophages, as key components of the innate immune system, playing a crucial role (Qiu et al., 2019; He et al., 2024). Previous studies showed that macrophages exhibit a spectrum of dynamic phenotypic alterations under infection (Yang et al., 2022; Gu et al., 2023). During inflammation, classically M1 macrophages are reprogrammed to the M2 phenotype, which contributes to the immunoparalysis in sepsis (Hotchkiss et al., 2013). Macrophages drive the primary immune response in the early stage of infection. Macrophages accumulate at the infection site during the early stages of *Staphylococcus aureus S. aureus*infection, and their numbers decrease in chronic states, which suggests a pivotal role for macrophages in the initial phase of infection (Li et al., 2024). In our research, we applied single-cell datasets and the hdWGCNA method to screen out immune cell types with the most ROS activity. In sepsis, macrophage was identified as one cell type with the most ROS activity in sepsis. This result proved that ROS are essential to macrophage bactericidal activity. Moreover, multiple machine learning methods (LASSO, SVM-RFE, Boruta, RF, and GBM) found out that LILRA5 mostly expressed in macrophage and that LILRA5^+^ macrophage had the most ROS activity. Our result validated the higher expression of LILRA5 in sepsis by two sepsis mouse models; moreover, after silencing LILRA5 expression in THP-1 cells, the OS activity was significant inhibited. Therefore, LILRA5 may potentially play an important role in ROS-mediated bacterial clearance.

LILRA5 is mostly expressed in monocytes and neutrophils. Hammoudeh et al. revealed LILRA5 has a potential tissue-specific immune signature in kidney under severe COVID-19 (Hammoudeh et al., 2021). LILRA5 triggers the innate immune response and plays a crucial role in inflammation regulation (Shi et al., 2024). Cross-linking of LILRA5 receptor induced secretion of IL-1β, IL-6, and TNF-α in monocytes, which accelerates the inflammation (Mitchell et al., 2008). A previous study identified LILRA5 as a marker gene in sepsis (Ning et al., 2023). Moreover, the host response to distinct bacterial infections (Gram-negative and Gram-positive) is different (Feezor et al., 2003). Another study proved that LILRA5 was a diagnosed marker in both *Escherichia coli*- and *S. aureus*-induced sepsis (Irani Shemirani, 2024). Studies of LILRA5 in macrophages are rare. In cardiac tissue, the HPA validation showed that LILRA5 expressed highly in macrophages, highlighting the importance of LILRA5 in cardiac immune response (Shi et al., 2024). Our study showed that LILRA5^+^ macrophage may play a vital role in OS in sepsis; however, its biological mechanism needs to be further studied. Interestingly, we also found that LILRA5 was highly expressed in the early stage of macrophage. This is consistent with a study of Willenborg et al. They found that a subpopulation of early-stage macrophages is characterized by mtROS production (Willenborg et al., 2021). These results demonstrated that LILRA5 may regulate OS of macrophage at an early stage.

In our study, we are also looking for an efficient method to identify cell clusters with the most OS. Previous studies on ROS mainly downloaded OS-related genes from GeneCards. However, not all genes exert an influence on OS in sepsis. Therefore, we applied the “Quartile method” rather than the “Median Method” in identifying cells with a higher OS activity. Cells were then defined as “high-OS cells”, “transition state”, and “low-OS cells”. DEGs between cell clusters with different OS states may make a real difference in sepsis-related OS activity.

Multiple studies have focused on screening biomarkers of sepsis. A previous study showed that S100A8/A9 were predictive markers based on serum samples of patients with sepsis (Chen et al., 2025). Given the important role of OS in biological processes in sepsis, our study focused on exploring hub genes and immune cells, which drive the OS in sepsis. A previous study showed that LILRA5 was a biomarker of sepsis (Ning et al., 2023). However, our study emphasized that screening out hub genes correlated with OS. Targeting LILRA5 may offer therapeutic benefits by modulating ROS activity in macrophages during early sepsis. However, because LILRA5 is also expressed in monocytes and neutrophils, systemic inhibition may disrupt broader immune functions. The lack of specific antagonists and the potential for immunosuppressive side effects pose substantial translational challenges. Future efforts should focus on developing selective inhibitors or antibody-based modulation strategies with cell-type specificity.

In our manuscript, we validated LILRA5 expression using two independent bulk RNA sepsis cohorts showing consistent upregulation in human sepsis whole blood. The dataset used by the research institute contains clinical information that does not provide the sepsis etiology (Gram-positive, Gram-negative, and fungal) of each sample. Therefore, the function of LILRA5^+^ macrophage varies by pathogen and is still unclear and, thus, needs further analysis. This study is limited by its reliance on publicly available datasets with relatively small sample sizes and potential inter-individual heterogeneity. Although batch correction and integration strategies were employed, some variability may persist. Additionally, while experimental validation was performed in THP-1 cells and murine models, the findings warrant further confirmation in primary human samples and prospective clinical cohorts. Moreover, we also performed functional validation in THP-1-derived macrophages, which is a well-established model for sepsis-related immunometabolic studies. Although our findings underwent robust validation across multiple datasets and the THP-1 model, the absence of validation using primary human macrophages may impact the direct clinical translation of our results. Future research should prioritize collaborative efforts to profile patient-derived macrophages at single-cell resolution, with a particular focus on LILRA5^+^ subsets in early sepsis. Moreover, our study focused on screening out LILRA5^+^ macrophage subsets with the most OS. The underlying mechanism of LILRA5-OS is still unclear and needs to be further studied.

## Conclusion

In conclusion, based on a multitude of bioinformatics and machine learning algorithms, we draw the single-cell landscape of dynamic changes of OS in sepsis. We found that the macrophage at the early stage had the most OS activity in sepsis and that LILRA5 may potentially be the gene regulating OS activity in this subtype of macrophage. Therefore, LILRA5 shows promise in regulating macrophage-mediated OS activity in sepsis.

## Data Availability

The datasets presented in this study can be found in online repositories. The names of the repository/repositories and accession number(s) can be found in the article/**Supplementary Material**.

## Supplementary material

Supplementary Figure 1 Preprocessing of scRNA-seq data. **(A)** The features, counts, percentages of mitochondrial, percentages of HB, and percentages of ribosome in each of the analyzed samples. **(B)** The scatter plot demonstrates the correlation between cell counts and intracellular gene counts, percentages of mitochondrial, percentages of HB, and percentages of ribosome following standardization.

Supplementary Figure 2 The density of each marker in each cluster. **(A)**: Monocyte, **(B)**: B cell, **(C)**: CD4^+^ T cell, **(D)**: Neutrophil, **(E)**: NK cell, **(F)** CD8^+^ T cell, **(G)**: Megakaryocyte, **(H)**: Macrophage, **(I)**: Mast cell, **(J)**: DC cell.

Supplementary Figure 3 The feature of each marker in each cluster. **(A)**: Monocyte, **(B)**: B cell, **(C)**: CD4^+^ T cell, **(D)**: Neutrophil, **(E)**: NK cell, **(F)** CD8^+^ T cell, **(G)**: Megakaryocyte, **(H)**: Macrophage, **(I)**: Mast cell, **(J)**: DC cell.

Supplementary Figure 4 The relationship between the marker genes of the 10 types of cells mentioned above, along with the relevant pathways enriched by GO analysis.

Supplementary Figure 5 The expression of 807 oxidative stress-related genes. **(A)** The distribution of 807 oxidative stress-related genes expressed in three different stages of sepsis. **(B)** The distribution of 807 oxidative stress-related genes expressed in ten types of cells.

Supplementary Figure 6 hdWGCNA analysis. **(A)** Ten gene modules were obtained and the top hub gene were presented according to the hdWGCNA pipeline. **(B)** Correlation analysis between each two gene modules. **(C)** The results of PPI analysis of the ten gene modules (black, blue, green, magenta, pink, and red).

Supplementary Figure 7 DO and GO analysis. **(A)** DO analysis conducted visualized by the boxplot. **(B)** GO analysis conducted visualized by the boxplot.

Supplementary Figure 8 Correlations between each gene and OS activity. **(A)** Correlation between each two genes and oxidative stress-related genes in the training dataset. **(B)** Correlation between each two genes and oxidative stress-related genes in the test dataset. **(C)** The OS activity score in control and sepsis samples of the training dataset. **(D**) The OS activity score in control and sepsis samples of the testing dataset. **(E)** Correlation of each four genes and oxidative stress gene set in the training dataset. **(F)** Correlation of each four genes and oxidative stress gene set in the test dataset. **(G)** Correlations between each genes and oxidative stress scores based on the single-cell sequencing datasets.

Supplementary Figure 9 The results of cellular communication indicated the quantity and intensity of cellular communication between LILRA5^+^ macrophage, LILRA5^-^ macrophage and other cell types.

Supplementary Figure 10 The ligand-receptor interactions between different cell types and LILRA5^-^ and LILRA5^+^ labeled macrophage.

## References

[B1] Bai B. Yang Y. Wang Q. Li M. Tian C. Liu Y.. (2020). NLRP3 inflammasome in endothelial dysfunction. Cell Death Dis. 11, 776. doi: 10.1038/s41419-020-02985-x, PMID: 32948742 PMC7501262

[B2] Baysoy A. Bai Z. Satija R. Fan R. (2023). The technological landscape and applications of single-cell multi-omics. Nat. Rev. Mol. Cell Biol. 24, 695–713. doi: 10.1038/s41580-023-00615-w, PMID: 37280296 PMC10242609

[B3] Chen J. Liu Z. Zhou F. Sun Y. Jiang Z. Zhao P. (2025). Assessment of S100A8/A9 and resistin as predictive biomarkers for mortality in critically ill patients with sepsis. Front. Cell Infect. Microbiol. 15, 1555307. doi: 10.3389/fcimb.2025.1555307, PMID: 40568710 PMC12188459

[B4] Chen S. Li Q. Shi H. Li F. Duan Y. Guo Q. (2024). New insights into the role of mitochondrial dynamics in oxidative stress-induced diseases. BioMed. Pharmacother. 178, 117084. doi: 10.1016/j.biopha.2024.117084, PMID: 39088967

[B5] Delano M. J. Ward P. A. (2016). The immune system’s role in sepsis progression, resolution, and long-term outcome. Immunol. Rev. 274, 330–353. doi: 10.1111/imr.12499, PMID: 27782333 PMC5111634

[B6] Feezor R. J. Oberholzer C. Baker H. V. Novick D. Rubinstein M. Moldawer L. L.. (2003). Molecular characterization of the acute inflammatory response to infections with gram-negative versus gram-positive bacteria. Infect. Immun. 71, 5803–5813. doi: 10.1128/IAI.71.10.5803-5813.2003, PMID: 14500502 PMC201043

[B7] Gu P. Liu R. Yang Q. Xie L. Wei R. Li J.. (2023). A metabolite from commensal Candida albicans enhances the bactericidal activity of macrophages and protects against sepsis. Cell Mol. Immunol. 20, 1156–1170. doi: 10.1038/s41423-023-01070-5, PMID: 37553429 PMC10541433

[B8] Gu J. Luo L. Wang Q. Yan S. Lin J. Li D.. (2018). Maresin 1 attenuates mitochondrial dysfunction through the ALX/cAMP/ROS pathway in the cecal ligation and puncture mouse model and sepsis patients. Lab. Invest. 98, 715–733. doi: 10.1038/s41374-018-0031-x, PMID: 29467458

[B9] Hammoudeh S. M. Hammoudeh A. M. Bhamidimarri P. M. Al Safar H. Mahboub B. Kunstner A.. (2021). Systems immunology analysis reveals the contribution of pulmonary and extrapulmonary tissues to the immunopathogenesis of severe COVID-19 patients. Front. Immunol. 12, 595150. doi: 10.3389/fimmu.2021.595150, PMID: 34262555 PMC8273737

[B10] He H. Zhang W. Jiang L. Tong X. Zheng Y. Xia Z. (2024). Endothelial cell dysfunction due to molecules secreted by macrophages in sepsis. Biomolecules 14 (8), 980. doi: 10.3390/biom14080980, PMID: 39199368 PMC11352357

[B11] Hotchkiss R. S. Monneret G. Payen D. (2013). Sepsis-induced immunosuppression: from cellular dysfunctions to immunotherapy. Nat. Rev. Immunol. 13, 862–874. doi: 10.1038/nri3552, PMID: 24232462 PMC4077177

[B12] Irani Shemirani M. (2024). Transcriptional markers classifying Escherichia coli and *Staphylococcus aureus* induced sepsis in adults: A data-driven approach. PloS One 19, e0305920. doi: 10.1371/journal.pone.0305920, PMID: 38968271 PMC11226107

[B13] Joffre J. Hellman J. (2021). Oxidative stress and endothelial dysfunction in sepsis and acute inflammation. Antioxid. Redox Signal. 35, 1291–1307. doi: 10.1089/ars.2021.0027, PMID: 33637016

[B14] Kuebart A. Gross K. Ripkens J. J. Tenge T. Raupach A. Schulz J.. (2023). Pravastatin Improves Colonic and Hepatic Microcirculatory Oxygenation during Sepsis without Affecting Mitochondrial Function and ROS Production in Rats. Int. J. Mol. Sci. 24 (6), 5455. doi: 10.3390/ijms24065455, PMID: 36982530 PMC10052315

[B15] Lai K. Song C. Gao M. Deng Y. Lu Z. Li N.. (2023). Uridine alleviates sepsis-induced acute lung injury by inhibiting ferroptosis of macrophage. Int. J. Mol. Sci. 24 (6). doi: 10.3390/ijms24065093, PMID: 36982166 PMC10049139

[B16] Li M. Wang B. Chen J. Jiang L. Zhou Y. Guo G.. (2024). *Staphylococcus aureus* SaeRS impairs macrophage immune functions through bacterial clumps formation in the early stage of infection. NPJ Biofilms Microbiomes 10, 102. doi: 10.1038/s41522-024-00576-8, PMID: 39370453 PMC11456606

[B17] Liu D. Huang S. Y. Sun J. H. Zhang H. C. Cai Q. L. Gao C.. (2022). Sepsis-induced immunosuppression: mechanisms, diagnosis and current treatment options. Mil. Med. Res. 9, 56. doi: 10.1186/s40779-022-00422-y, PMID: 36209190 PMC9547753

[B18] Mitchell A. Rentero C. Endoh Y. Hsu K. Gaus K. Geczy C.. (2008). LILRA5 is expressed by synovial tissue macrophages in rheumatoid arthritis, selectively induces pro-inflammatory cytokines and IL-10 and is regulated by TNF-alpha, IL-10 and IFN-gamma. Eur. J. Immunol. 38, 3459–3473. doi: 10.1002/eji.200838415, PMID: 19009525

[B19] Neri M. Riezzo I. Pomara C. Schiavone S. Turillazzi E. (2016). Oxidative-nitrosative stress and myocardial dysfunctions in sepsis: evidence from the literature and postmortem observations. Mediators Inflamm. 2016, 3423450. doi: 10.1155/2016/3423450, PMID: 27274621 PMC4870364

[B20] Ning J. Fan X. Sun K. Wang X. Li H. Jia K.. (2023). Single-cell sequence analysis combined with multiple machine learning to identify markers in sepsis patients: LILRA5. Inflammation 46, 1236–1254. doi: 10.1007/s10753-023-01803-8, PMID: 36920635

[B21] Ouyang J. Hong Y. Wan Y. He X. Geng B. Yang X.. (2024). PVB exerts anti-inflammatory effects by inhibiting the activation of MAPK and NF-kappaB signaling pathways and ROS generation in neutrophils. Int. Immunopharmacol. 126, 111271. doi: 10.1016/j.intimp.2023.111271, PMID: 38006749

[B22] Park S. Juliana C. Hong S. Datta P. Hwang I. Fernandes-Alnemri T.. (2013). The mitochondrial antiviral protein MAVS associates with NLRP3 and regulates its inflammasome activity. J. Immunol. 191, 4358–4366. doi: 10.4049/jimmunol.1301170, PMID: 24048902 PMC3848201

[B23] Qiu P. Liu Y. Zhang J. (2019). Review: the role and mechanisms of macrophage autophagy in sepsis. Inflammation 42, 6–19. doi: 10.1007/s10753-018-0890-8, PMID: 30194660

[B24] Sanlioglu S. Williams C. M. Samavati L. Butler N. S. Wang G. McCray P. B. Jr.. (2001). Lipopolysaccharide induces Rac1-dependent reactive oxygen species formation and coordinates tumor necrosis factor-alpha secretion through IKK regulation of NF-kappa B. J. Biol. Chem. 276, 30188–30198. doi: 10.1074/jbc.M102061200, PMID: 11402028

[B25] Shi K. Chen X. Zhao Y. Li P. Chai J. Qiu J.. (2024). Identification of potential therapeutic targets for nonischemic cardiomyopathy in European ancestry: an integrated multiomics analysis. Cardiovasc. Diabetol. 23, 338. doi: 10.1186/s12933-024-02431-8, PMID: 39267096 PMC11396958

[B26] Shi X. Tan S. Tan S. (2021). NLRP3 inflammasome in sepsis (Review). Mol. Med. Rep. 24 (1), 514. doi: 10.3892/mmr.2021.12153, PMID: 33982766

[B27] Street K. Risso D. Fletcher R. B. Das D. Ngai J. Yosef N.. (2018). Slingshot: cell lineage and pseudotime inference for single-cell transcriptomics. BMC Genomics 19, 477. doi: 10.1186/s12864-018-4772-0, PMID: 29914354 PMC6007078

[B28] Vera S. Martinez R. Gormaz J. G. Gajardo A. Galleguillos F. Rodrigo R. (2015). Novel relationships between oxidative stress and angiogenesis-related factors in sepsis: New biomarkers and therapies. Ann. Med. 47, 289–300. doi: 10.3109/07853890.2015.1029967, PMID: 25998489

[B29] Vincent J. L. (2022). Current sepsis therapeutics. EBioMedicine 86, 104318. doi: 10.1016/j.ebiom.2022.104318, PMID: 36470828 PMC9782815

[B30] Wang F. Chen M. Ma J. Wang C. Wang J. Xia H.. (2022). Integrating bulk and single-cell sequencing reveals the phenotype-associated cell subpopulations in sepsis-induced acute lung injury. Front. Immunol. 13, 981784. doi: 10.3389/fimmu.2022.981784, PMID: 36405762 PMC9666384

[B31] West A. P. Brodsky I. E. Rahner C. Woo D. K. Erdjument-Bromage H. Tempst P.. (2011). TLR signalling augments macrophage bactericidal activity through mitochondrial ROS. Nature 472, 476–480. doi: 10.1038/nature09973, PMID: 21525932 PMC3460538

[B32] Willenborg S. Sanin D. E. Jais A. Ding X. Ulas T. Nuchel J.. (2021). Mitochondrial metabolism coordinates stage-specific repair processes in macrophages during wound healing. Cell Metab. 33, 2398–414 e9. doi: 10.1016/j.cmet.2021.10.004, PMID: 34715039

[B33] Xu X. Pang Y. Fan X. (2025). Mitochondria in oxidative stress, inflammation and aging: from mechanisms to therapeutic advances. Signal Transduct Target Ther. 10, 190. doi: 10.1038/s41392-025-02253-4, PMID: 40500258 PMC12159213

[B34] Xu P. Tao Z. Zhang C. (2024). Integrated multi-omics and artificial intelligence to explore new neutrophils clusters and potential biomarkers in sepsis with experimental validation. Front. Immunol. 15, 1377817. doi: 10.3389/fimmu.2024.1377817, PMID: 38868781 PMC11167131

[B35] Yang G. Cheng J. Xu J. Shen C. Lu X. He C.. (2024). Metabolic heterogeneity in clear cell renal cell carcinoma revealed by single-cell RNA sequencing and spatial transcriptomics. J. Transl. Med. 22, 210. doi: 10.1186/s12967-024-04848-x, PMID: 38414015 PMC10900752

[B36] Yang K. Fan M. Wang X. Xu J. Wang Y. Tu F.. (2022). Lactate promotes macrophage HMGB1 lactylation, acetylation, and exosomal release in polymicrobial sepsis. Cell Death Differ. 29, 133–146. doi: 10.1038/s41418-021-00841-9, PMID: 34363018 PMC8738735

[B37] Zhang W. Y. Chen Z. H. An X. X. Li H. Zhang H. L. Wu S. J.. (2023). Analysis and validation of diagnostic biomarkers and immune cell infiltration characteristics in pediatric sepsis by integrating bioinformatics and machine learning. World J. Pediatr. 19, 1094–1103. doi: 10.1007/s12519-023-00717-7, PMID: 37115484 PMC10533616

