# Supplementary figures and images for: LILRA5+ macrophages drive early oxidative stress surge in sepsis: a single-cell transcriptomic landscape with therapeutic implications
Source: Front Cell Infect Microbiol. 2025 Jul 28;15:1606401. doi: 10.3389/fcimb.2025.1606401 (PMC12336265; doi:10.3389/fcimb.2025.1606401)

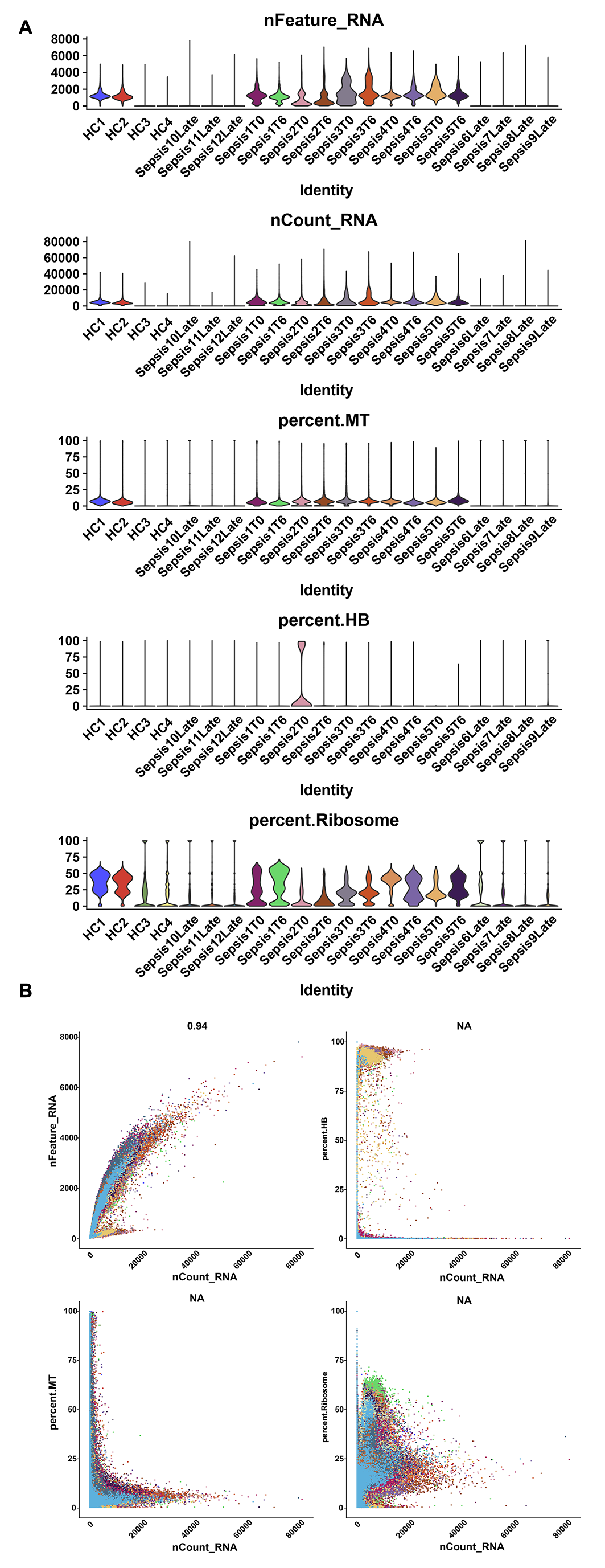

Supplement: Supplementary Figure 1 — Preprocessing of scRNA-seq data. (A) The features, counts, percentages of mitochondrial, percentages of HB, and percentages of ribosome in each of the analyzed samples. (B) The scatter plot demonstrates the correlation between cell counts and intracellular gene counts, percentages of mitochondrial, percentages of HB, and percentages of ribosome following standardization. [file Image1.tif]

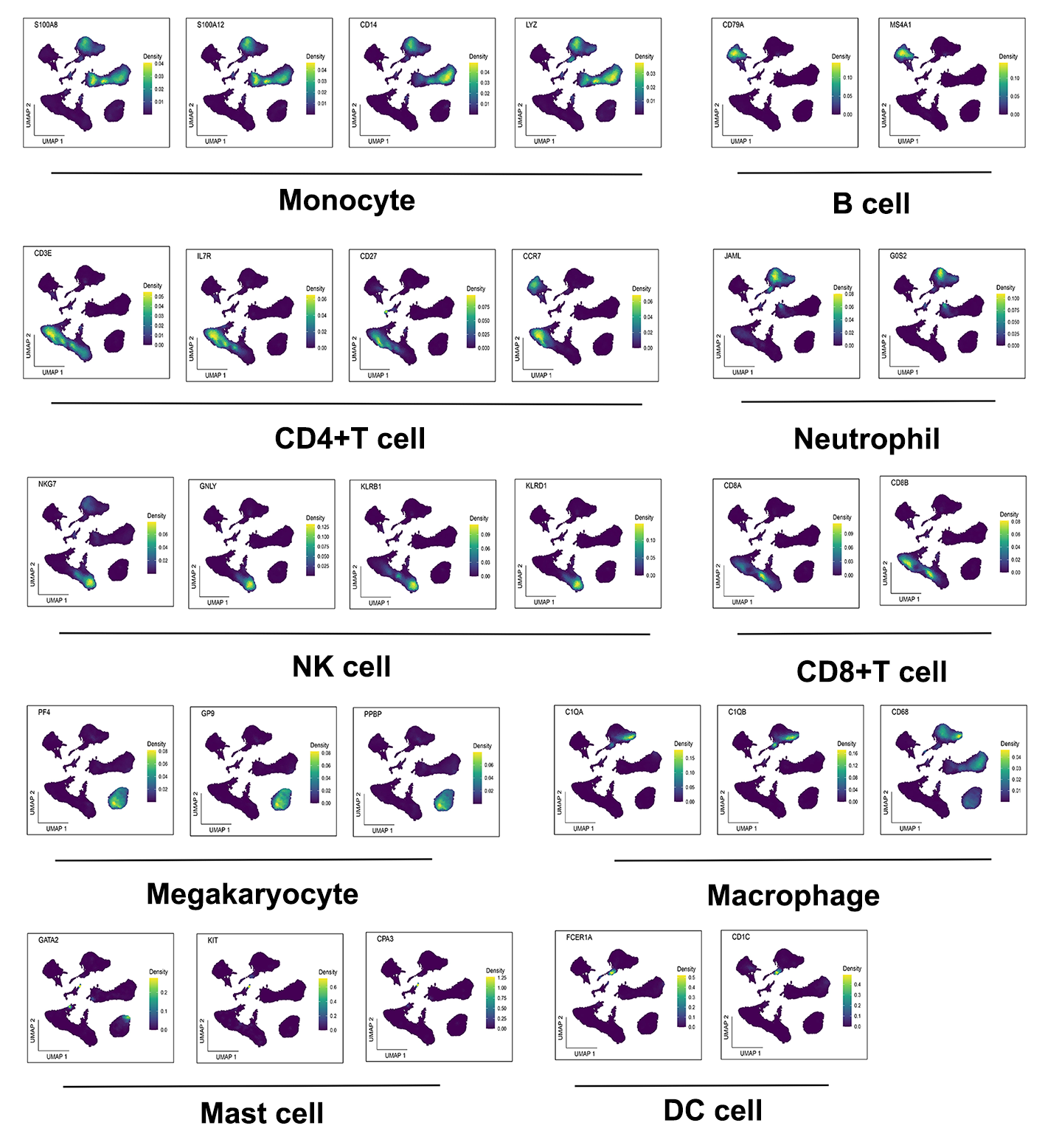

Supplement: Supplementary Figure 2 — The density of each marker in each cluster. (A): Monocyte, (B): B cell, (C): CD4+ T cell, (D): Neutrophil, (E): NK cell, (F) CD8+ T cell, (G): Megakaryocyte, (H): Macrophage, (I): Mast cell, (J): DC cell. [file Image2.tif]

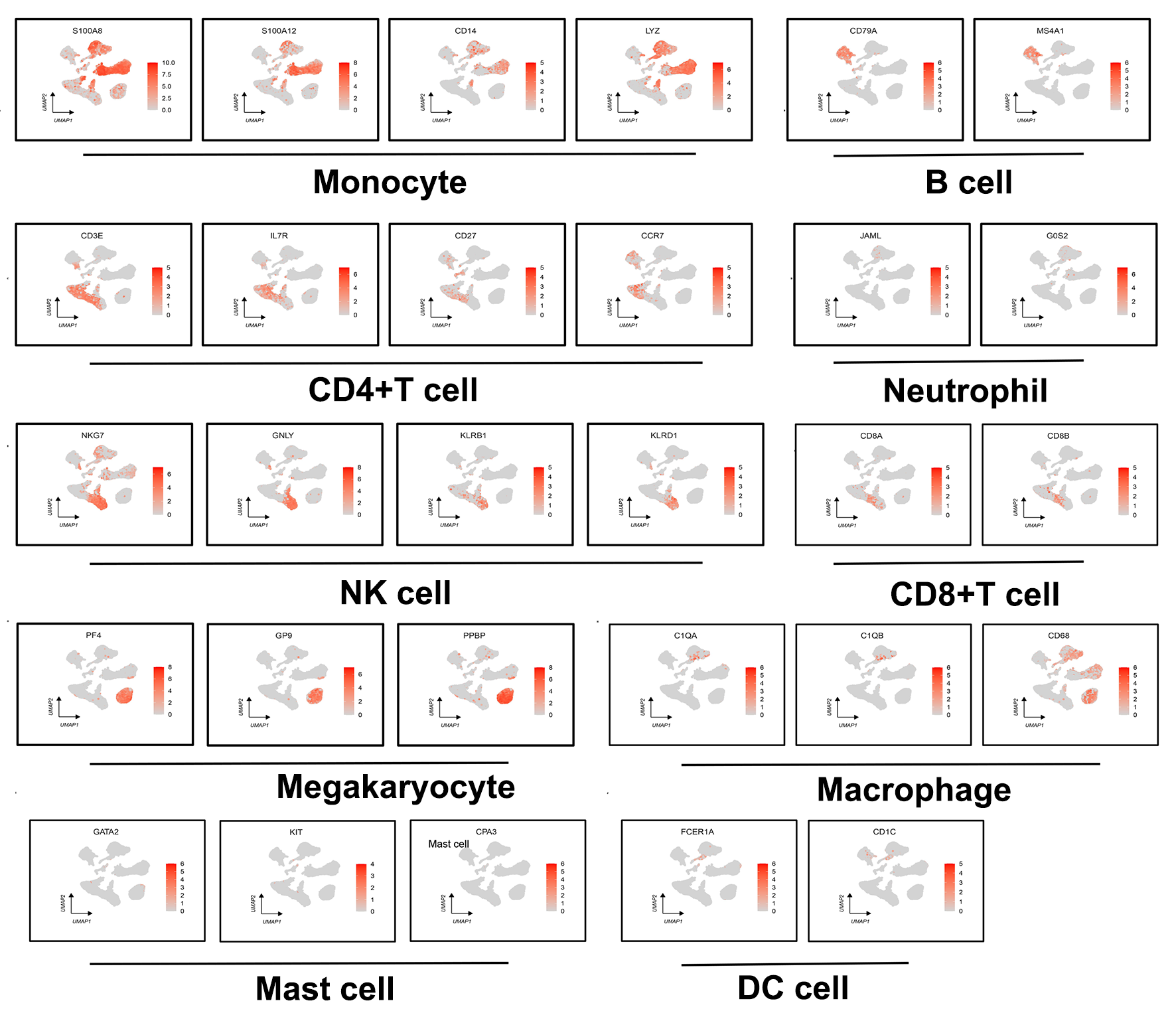

Supplement: Supplementary Figure 3 — The feature of each marker in each cluster. (A): Monocyte, (B): B cell, (C): CD4+ T cell, (D): Neutrophil, (E): NK cell, (F) CD8+ T cell, (G): Megakaryocyte, (H): Macrophage, (I): Mast cell, (J): DC cell. [file Image3.tif]

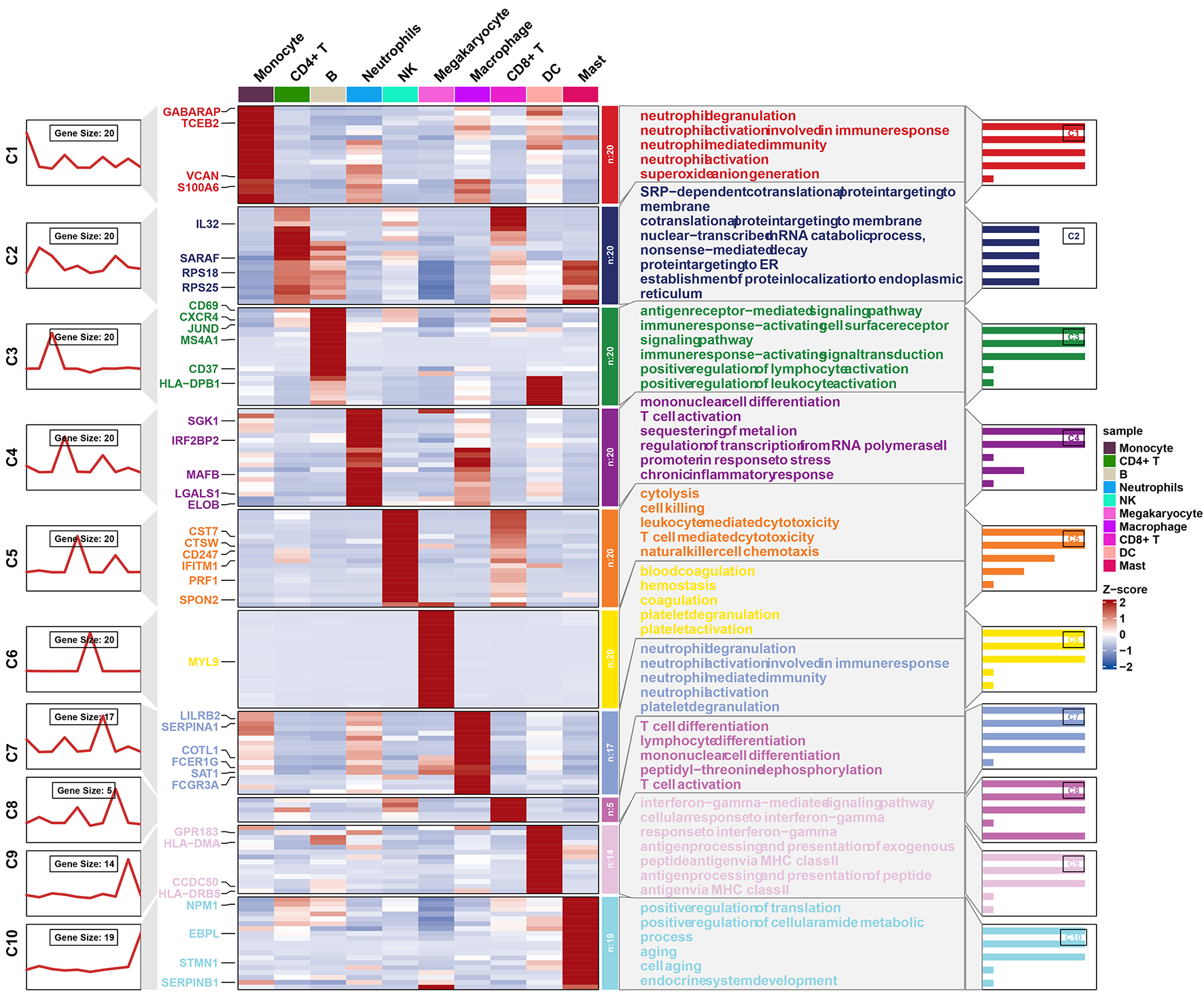

Supplement: Supplementary Figure 4 — The relationship between the marker genes of the 10 types of cells mentioned above, along with the relevant pathways enriched by GO analysis. [file Image4.tif]

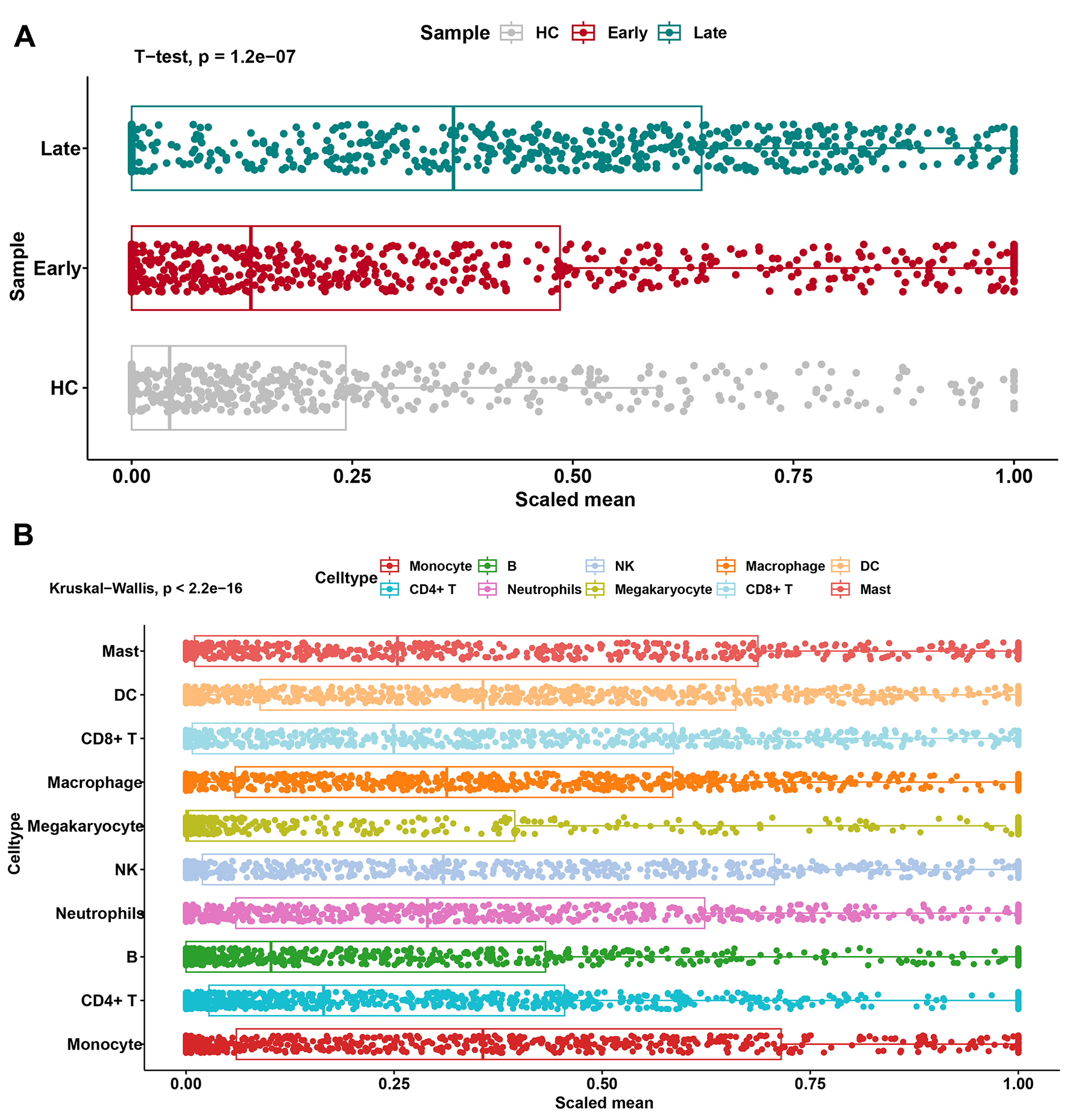

Supplement: Supplementary Figure 5 — The expression of 807 oxidative stress-related genes. (A) The distribution of 807 oxidative stress-related genes expressed in three different stages of sepsis. (B) The distribution of 807 oxidative stress-related genes expressed in ten types of cells. [file Image5.tif]

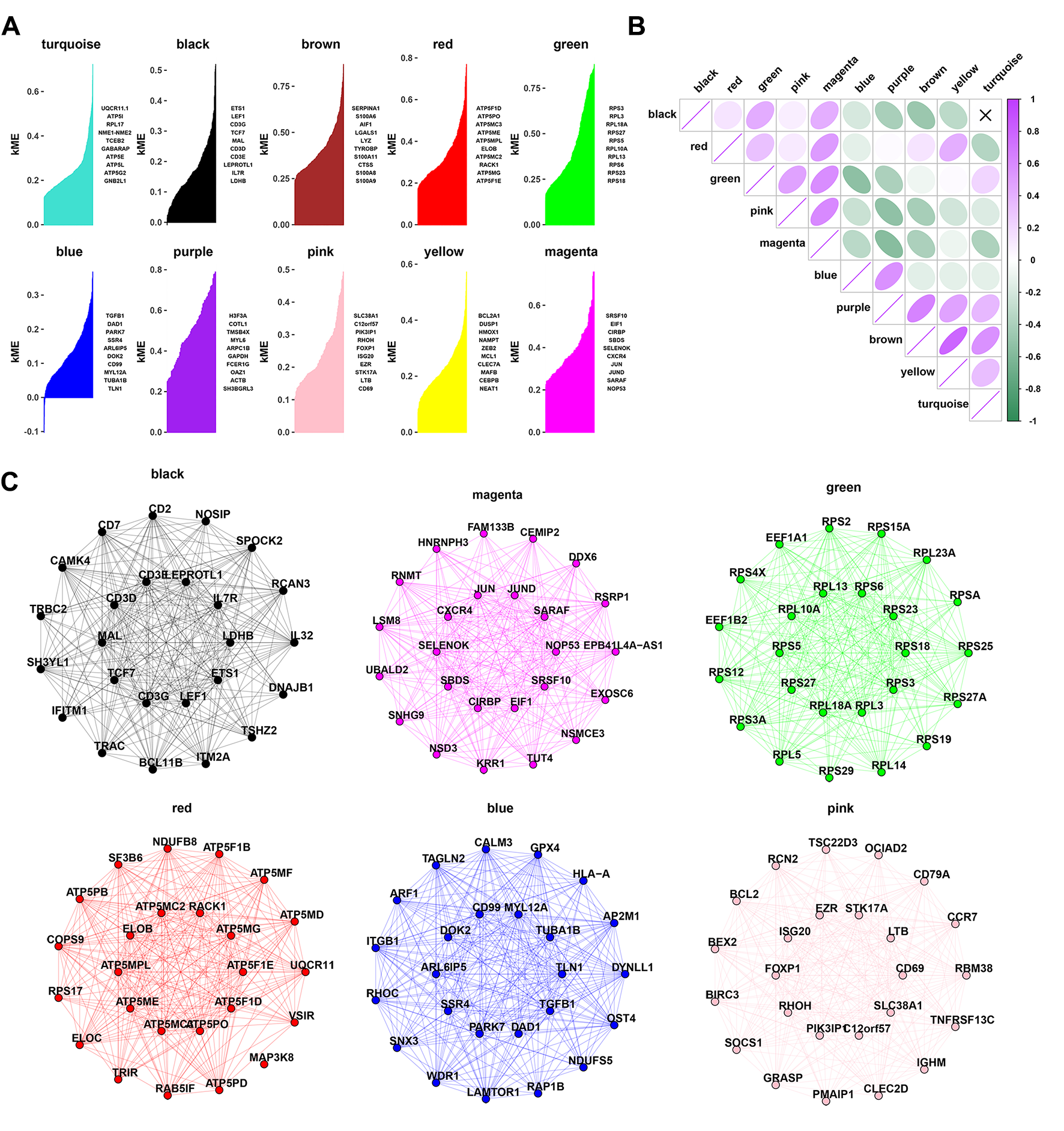

Supplement: Supplementary Figure 6 — hdWGCNA analysis. (A) Ten gene modules were obtained and the top hub gene were presented according to the hdWGCNA pipeline. (B) Correlation analysis between each two gene modules. (C) The results of PPI analysis of the ten gene modules (black, blue, green, magenta, pink, and red). [file Image6.tif]

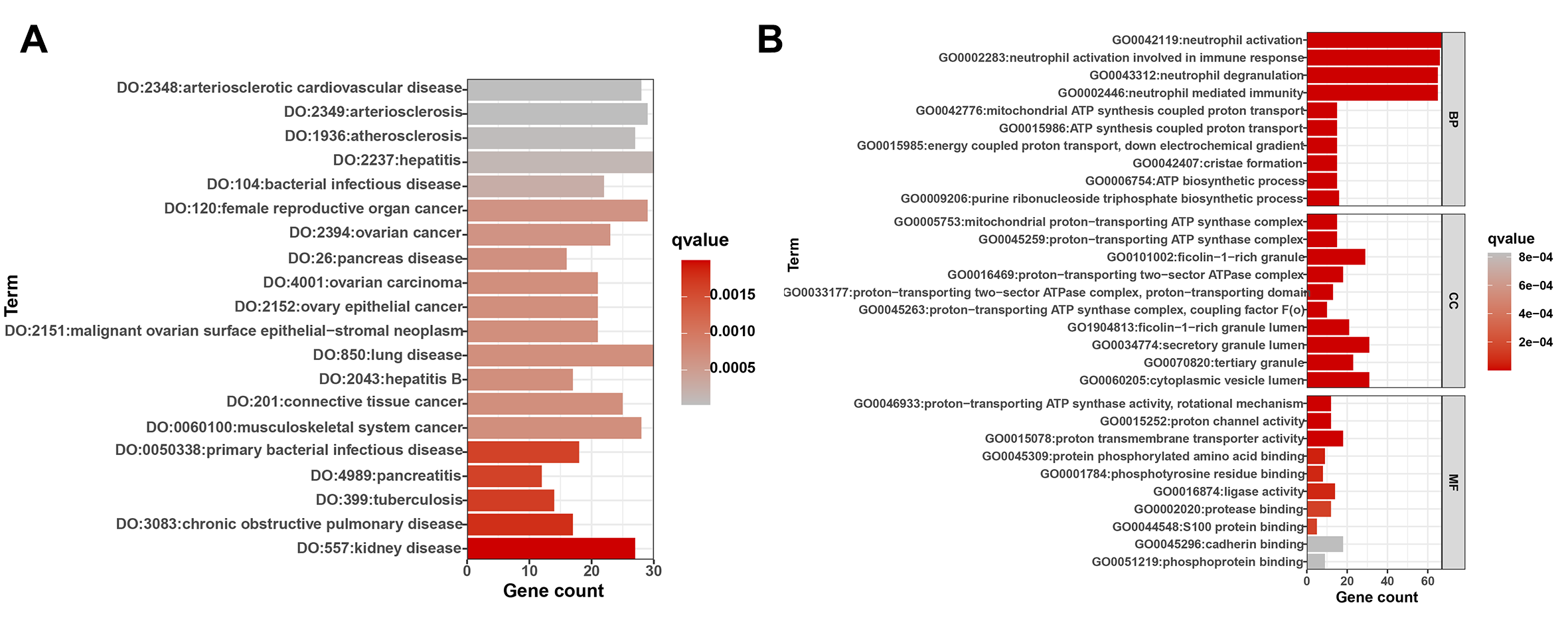

Supplement: Supplementary Figure 7 — DO and GO analysis. (A) DO analysis conducted visualized by the boxplot. (B) GO analysis conducted visualized by the boxplot. [file Image7.tif]

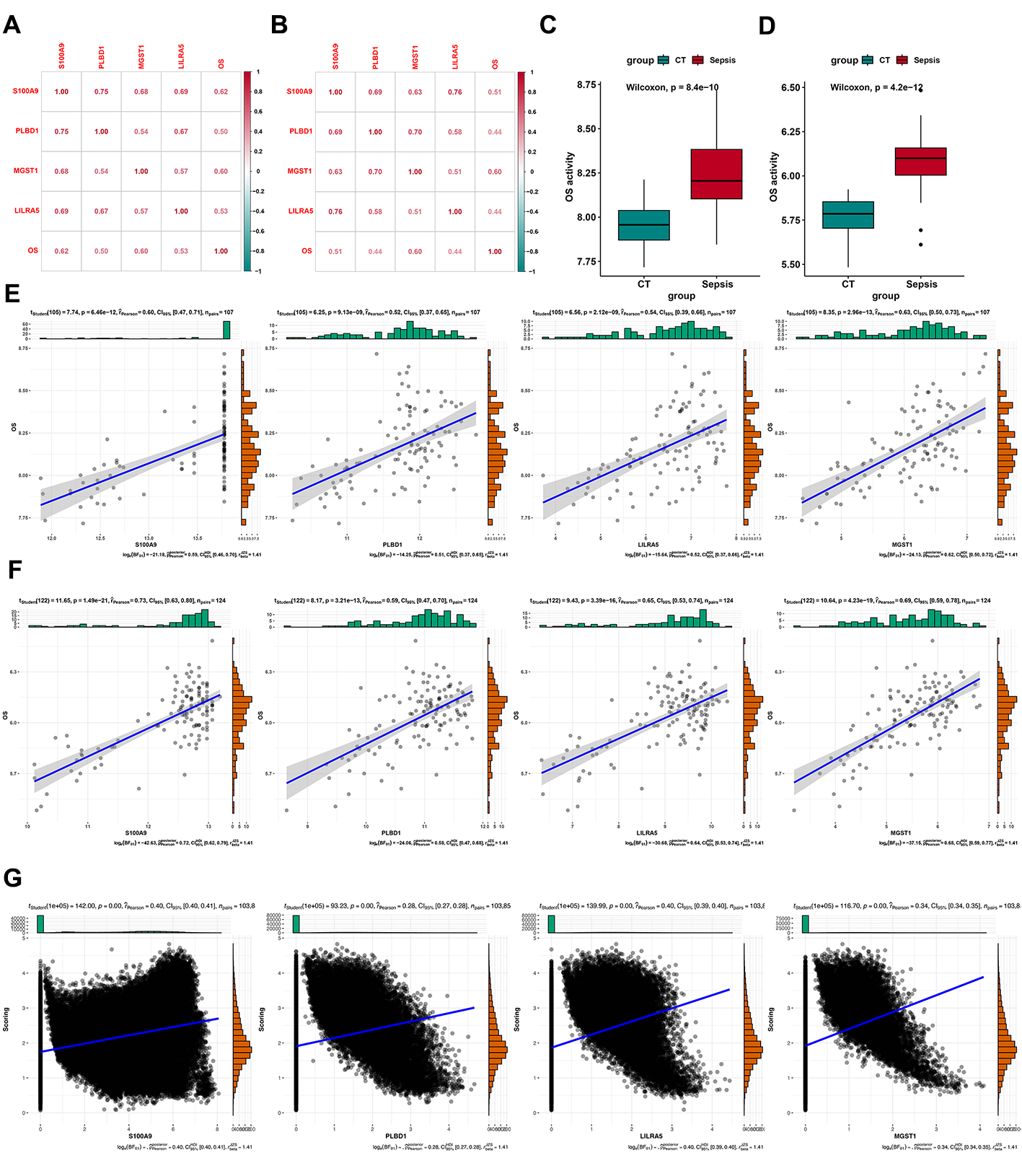

Supplement: Supplementary Figure 8 — Correlations between each gene and OS activity. (A) Correlation between each two genes and oxidative stress-related genes in the training dataset. (B) Correlation between each two genes and oxidative stress-related genes in the test dataset. (C) The OS activity score in control and sepsis samples of the training dataset. (D) The OS activity score in control and sepsis samples of the testing dataset. (E) Correlation of each four genes and oxidative stress gene set in the training dataset. (F) Correlation of each four genes and oxidative stress gene set in the test dataset. (G) Correlations between each genes and oxidative stress scores based on the single-cell sequencing datasets. [file Image8.tif]

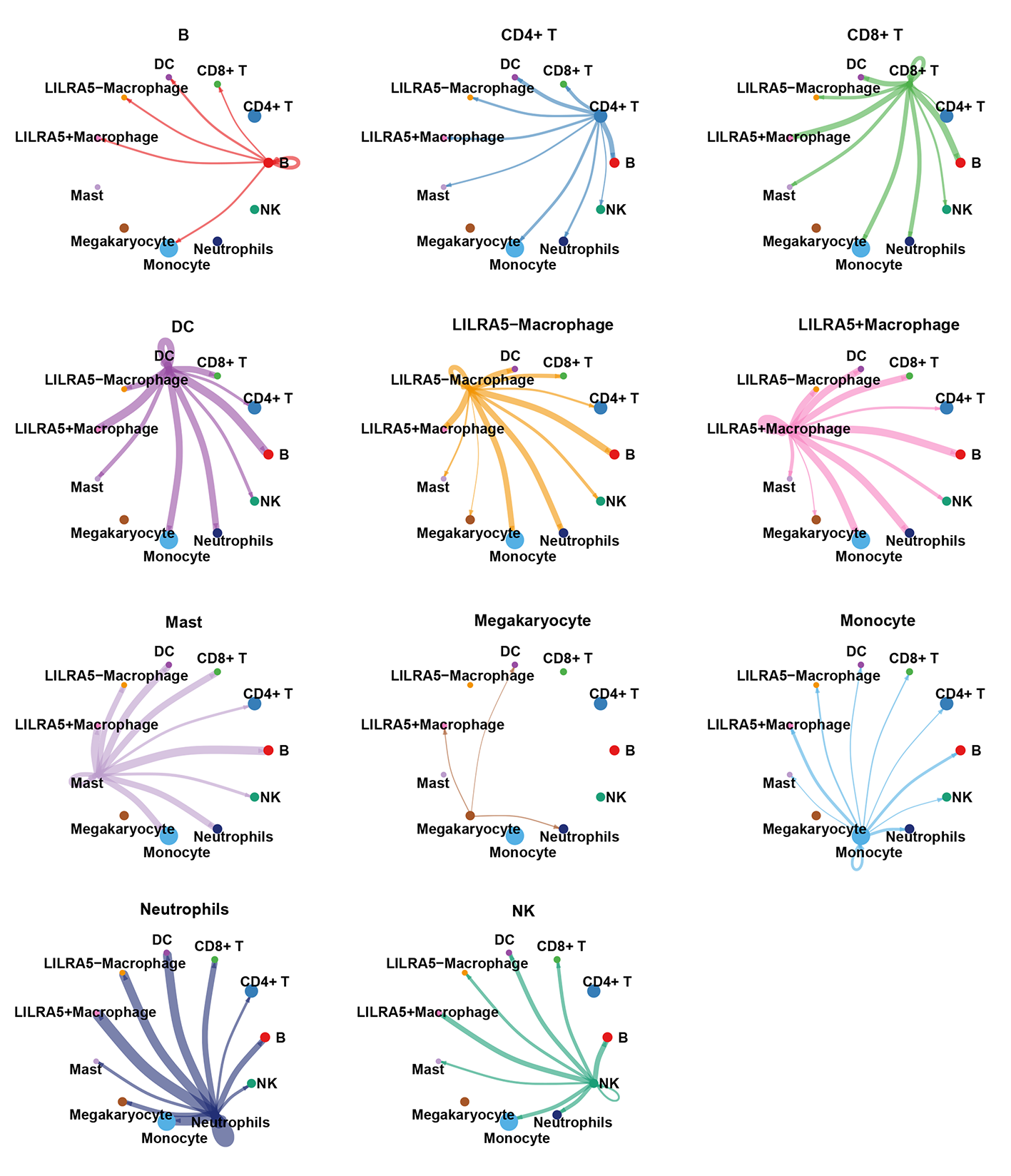

Supplement: Supplementary Figure 9 — The results of cellular communication indicated the quantity and intensity of cellular communication between LILRA5+ macrophage, LILRA5- macrophage and other cell types. [file Image9.tif]

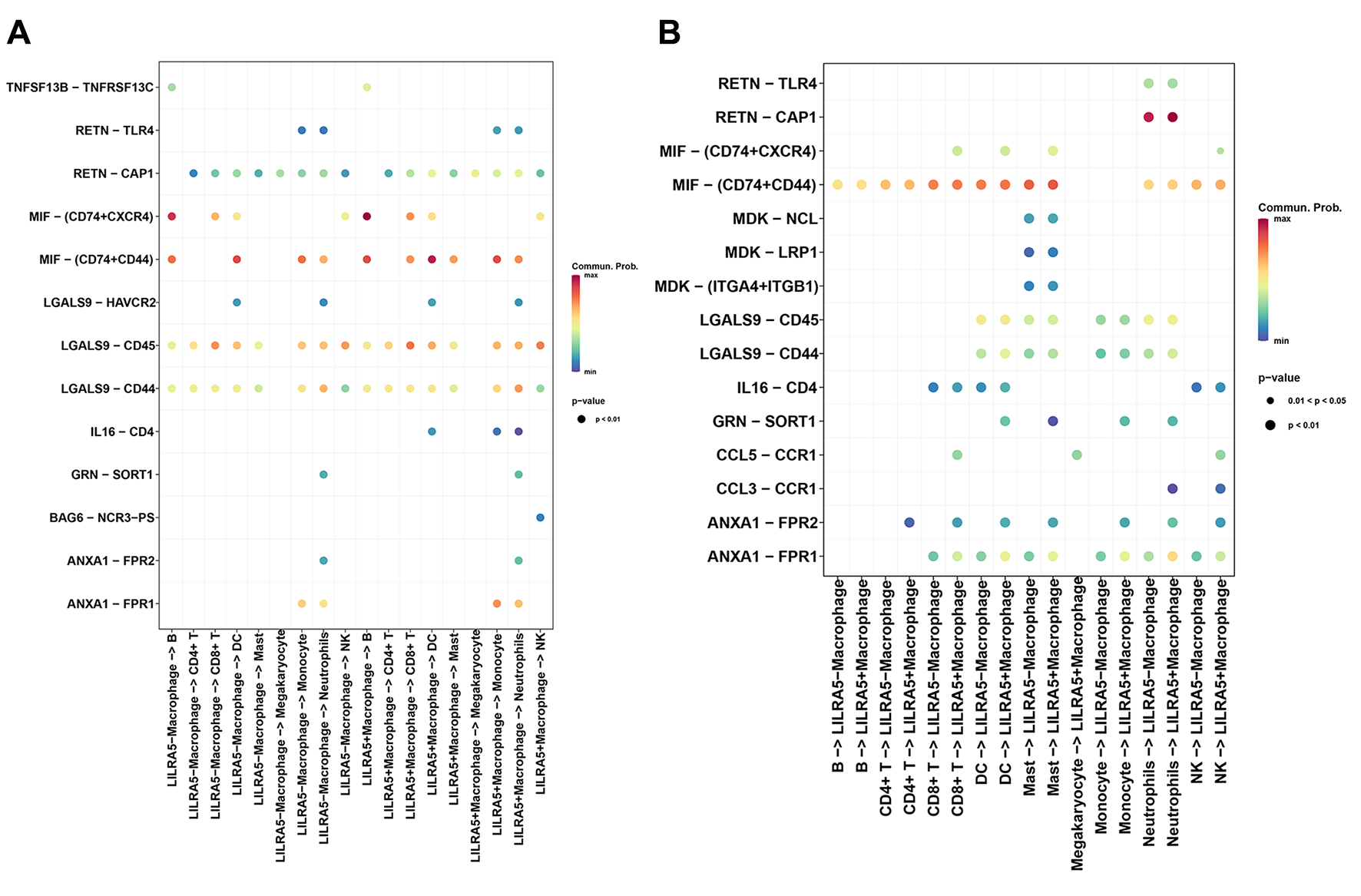

Supplement: Supplementary Figure 10 — The ligand-receptor interactions between different cell types and LILRA5- and LILRA5+ labeled macrophage. [file Image10.tif]
