# Supplementary material for: LILRA5+ macrophages drive early oxidative stress surge in sepsis: a single-cell transcriptomic landscape with therapeutic implications
Source: Front Cell Infect Microbiol. 2025 Jul 28;15:1606401. doi: 10.3389/fcimb.2025.1606401 (PMC12336265; doi:10.3389/fcimb.2025.1606401)
Supplement: Supplementary file 11 [file Table1.docx]

**Supplementary Table 1.Details of the GSE167363 dataset.**

| **Dataset** | | GSE167363 |
| --- | --- | --- |
| **Status** | | Public on Mar 03, 2021 |
| **Organism** | | Homo sapiens |
| **Experiment type** | | Expression profiling by high throughput sequencing |
| **Overall design** | | Single-cell RNA-sequencing of human peripheral blood mononuclear cells from heathy controls, survivor and non-survivor of gram-negative sepsis patients. |
| **Platforms** | | GPL24676 Illumina NovaSeq 6000 (Homo sapiens) |
| **Samples** | | |
| 1 | GSM5102900 | Nonsepsis control 1 (HC1) |
| 2 | GSM5102901 | Nonsepsis control 2 (HC2) |
| 3 | GSM5102902 | Nonsurvivor late stage (NS LS) T0 |
| 4 | GSM5102903 | Nonsurvivor late stage (NS LS) T6 |
| 5 | GSM5102904 | Sepsis survivor 1 (S1) T0 |
| 6 | GSM5102905 | Sepsis survivor 1 (S1) T6 |
| 7 | GSM5511351 | Nonsurvivor early stage (NS ES) T0 |
| 8 | GSM5511352 | Nonsurvivor early stage (NS ES) T6 |
| 9 | GSM5511353 | Sepsis survivor 2 (S2) T0 |
| 10 | GSM5511354 | Sepsis survivor 2 (S2) T6 |
| 11 | GSM5511355 | Sepsis survivor 3 (S3) T0 |
| 12 | GSM5511356 | Sepsis survivor 3 (S3) T6 |
