# Supplementary material for: LILRA5+ macrophages drive early oxidative stress surge in sepsis: a single-cell transcriptomic landscape with therapeutic implications
Source: Front Cell Infect Microbiol. 2025 Jul 28;15:1606401. doi: 10.3389/fcimb.2025.1606401 (PMC12336265; doi:10.3389/fcimb.2025.1606401)
Supplement: Supplementary file 12 [file Table2.docx]

**Supplementary Table 2.Details of the GSE17543 dataset.**

| **Dataset** | | GSE175453 |
| --- | --- | --- |
| **Status** | | Public on Jun 04, 2021 |
| **Organism** | | Homo sapiens |
| **Experiment type** | | Expression profiling by high throughput sequencing |
| **Overall design** | | A mixture of whole blood myeloid-enriched and Ficoll-enriched peripheral blood mononuclear cells from four late septic patients (post-sepsis day 14-21, RAP n=1 and CCI n=3) and five healthy subjects underwent Cellular Indexing of Transcriptomes and Epitopes by Sequencing (CITE-seq). |
| **Platforms** | | GPL18573 Illumina NextSeq 500 (Homo sapiens)  GPL24676 Illumina NovaSeq 6000 (Homo sapiens) |
| **Samples** | |  |
| 1 | GSM5333784 | Healthy Control 1 |
| 2 | GSM5333785 | Healthy Control 2 |
| 3 | GSM5333786 | Sepsis 1 |
| 4 | GSM5333787 | Sepsis 2 |
| 5 | GSM5333788 | Sepsis 3 |
| 6 | GSM5333789 | Sepsis 4 |
| 7 | GSM5333790 | Healthy Control 3 |
| 8 | GSM5333791 | Healthy Control 4 |
| 9 | GSM5333792 | Healthy Control 5 |
