# Supplementary material for: LILRA5+ macrophages drive early oxidative stress surge in sepsis: a single-cell transcriptomic landscape with therapeutic implications
Source: Front Cell Infect Microbiol. 2025 Jul 28;15:1606401. doi: 10.3389/fcimb.2025.1606401 (PMC12336265; doi:10.3389/fcimb.2025.1606401)
Supplement: Supplementary file 13 [file Table3.docx]

**Supplementary Table 3. Details of the GSE57065 dataset.**

| **Dataset** | GSE57065 |
| --- | --- |
| **Status** | Public on Nov 05, 2014 |
| **Organism** | Homo sapiens |
| **Experiment type** | Expression profiling by array |
| **Overall design** | Twenty-eight ICU patients were enrolled at the onset of septic shock. Blood samples were collected within 30 minutes, 24 and 48 hours after septic shock and compared to twenty-five healthy volunteers |
| **Platforms** | GPL570 [HG-U133_Plus_2] Affymetrix Human Genome U133 Plus 2.0 Array |
