# Supplementary material for: LILRA5+ macrophages drive early oxidative stress surge in sepsis: a single-cell transcriptomic landscape with therapeutic implications
Source: Front Cell Infect Microbiol. 2025 Jul 28;15:1606401. doi: 10.3389/fcimb.2025.1606401 (PMC12336265; doi:10.3389/fcimb.2025.1606401)
Supplement: Supplementary file 14 [file Table4.docx]

**Supplementary Table 4.Details of the GSE95233 dataset.**

| **Dataset** | GSE95233 |
| --- | --- |
| **Status** | Public on Nov 27, 2017 |
| **Organism** | Homo sapiens |
| **Experiment type** | Expression profiling by array |
| **Overall design** | 51 septic shock patients and 22 healthy volunteers were included in this study. Septic shock patients were sampled twice, at admission, and a second time at D2 or D3. Admission samples from septic shock patients were compared to healthy volunteers, and according to day 28 survival status. Modulation of gene expression between the 2 time points was also analyzed according to day 28 survival. |
| **Platforms** | GPL570 [HG-U133_Plus_2] Affymetrix Human Genome U133 Plus 2.0 Array |
